# The PROGAIN trial: A randomized controlled trial of high-protein peripheral parenteral nutrition on nitrogen balance and recovery after gastric cancer surgery — study protocol

**DOI:** 10.1371/journal.pone.0355677

**Published:** 2026-08-07

**Authors:** Jong Hyuk Yun, MinJee Han, GaRam Bae, Hyunsub Shin, GeumJong Song, MyoungWon Son

**Affiliations:** 1 Department of Surgery, Soonchunhyang Cheonan Hospital, Soonchunhyang University College of Medicine, Cheonan, Republic of Korea; 2 Department of Clinical Nutrition, Soonchunhyang Cheonan Hospital, Soonchunhyang University College of Medicine, Cheonan, Republic of Korea; 3 Department of Pharmacy, Soonchunhyang Cheonan Hospital, Soonchunhyang University College of Medicine, Cheonan, Republic of Korea; Athens Medical Group, Psychiko Clinic, GREECE

## Abstract

**Introduction:**

Patients undergoing curative gastrectomy for gastric cancer are vulnerable to perioperative protein loss, negative nitrogen balance and delayed recovery. Conventional peripheral parenteral nutrition (PN) formulations may not provide sufficient protein at clinically practical infusion volumes, and whether high-protein (amino acid–enriched) PN improves postoperative nitrogen balance after gastrectomy has not been tested in a randomized controlled trial. The PROGAIN trial evaluates the effect of perioperative high-protein PN on nitrogen balance and recovery outcomes in this population.

**Methods and analysis:**

PROGAIN is a single-center, randomized, open-label, parallel-group superiority trial with blinded laboratory assessment and coded-group analysis of the primary endpoint. The trial will enroll 110 patients with gastric cancer scheduled for curative gastrectomy. Participants are allocated 1:1 to high-protein PN or conventional PN, administered through a peripheral intravenous line on the day before surgery and postoperative day (POD) 1 through POD 5. PN is delivered at a daily caloric target of 20 kcal/kg/day using a stepwise schedule informed by an institutional retrospective audit of oral intake. The primary outcome is nitrogen balance on POD 5, calculated using a simplified Blackburn equation. Secondary outcomes include nutritional indices, skeletal muscle index, glycemic control, postoperative complications classified by Clavien–Dindo grade and functional recovery measures. The primary analysis will compare the two arms using a two-sample t-test in the intention-to-treat population. The trial has 80% power to detect a between-group mean difference of 3 g/day at a two-sided α of 0.05.

**Ethics and dissemination:**

The trial is approved by the Institutional Review Board of Soonchunhyang Cheonan Hospital and conducted in accordance with the Declaration of Helsinki and ICH–Good Clinical Practice. Findings will be disseminated through peer-reviewed publication, scientific meetings and results reporting on ClinicalTrials.gov.

**Trial registration:**

ClinicalTrials.gov, NCT07488611.

## Introduction

Gastric cancer is a leading cause of cancer-related morbidity and mortality worldwide and is highly prevalent in East Asia [1]; in the Republic of Korea, it is the fourth most common cancer [2]. Although gastrectomy is the cornerstone of curative treatment, it substantially alters digestive anatomy and physiology, reduces oral intake and nutrient absorption, and provokes a systemic surgical stress response that contributes to marked perioperative deterioration in nutritional status.

Cancer-related malnutrition affects approximately 41% of patients globally and is associated with a substantially increased risk of mortality and postoperative complications [3]. The early postoperative period after gastrectomy is characterized by an acute catabolic state, peaking within the first 48–72 hours, in which accelerated whole-body protein breakdown produces a markedly negative nitrogen balance and progressive depletion of skeletal muscle mass [4,5]. Inadequate perioperative protein supply is linked to delayed wound healing, infectious complications, prolonged hospital stay, and impaired tolerance of adjuvant chemotherapy [6,7] — consequences particularly relevant in gastric cancer surgery, where postoperative oral intake is restricted and highly variable between patients, yet the actual protein delivery achieved during this period has not been prospectively quantified. Our institutional experience has likewise highlighted the clinical importance of postoperative nutritional status after gastrectomy in patients with gastric cancer [8,9]. These findings position perioperative nutritional state as a modifiable determinant of the gastric cancer treatment trajectory.

To attenuate the catabolic response and support recovery, current European Society for Clinical Nutrition and Metabolism (ESPEN) cancer guidelines recommend a daily protein intake of at least 1.0 g/kg, increasing to 1.5 g/kg or higher in patients with sarcopenia, advanced age, or established malnutrition [7]; the 2025 update to the ESPEN guideline on clinical nutrition in surgery reaffirms perioperative protein supplementation as a central component of recovery-oriented care [10]. Among older adults and critically ill patients, evidence further supports protein delivery up to 2.0 g/kg/day to preserve lean body mass [11,12]. However, conventional peripheral parenteral nutrition (PN) formulations were not designed to meet these targets at clinically practical infusion volumes. High-protein (amino acid–enriched) PN formulations have been developed to deliver more nitrogen at the same daily volume, with a correspondingly lower non-protein calorie-to-nitrogen ratio (Table 2). At the trial’s caloric target, conventional PN provides approximately 0.9 g/kg/day of protein equivalent from amino acids (below the ESPEN minimum of 1.0 g/kg/day), whereas high-protein PN provides approximately 1.1 g/kg/day, thereby bringing protein delivery within the ESPEN-recommended range (1.0–1.5 g/kg/day) while remaining feasible via peripheral venous access [7,10].

Despite this rationale, prospective evidence for the clinical effectiveness of high-protein PN in patients undergoing gastrectomy is lacking. Trials in critically ill patients have demonstrated that high-protein nutritional support improves measures of protein metabolism [12]; observational data after oesophagectomy suggest that protein-enhanced feeds attenuate postoperative catabolism [13]; a randomized study of arginine-enriched early enteral nutrition has shown improvements in nitrogen balance after total gastrectomy [14]; and a systematic review of high-protein home PN has documented favorable effects in oncology patients [15]. Although these studies support the biological plausibility of enhanced protein delivery, their populations, routes of nutrition, and clinical contexts differ substantially from perioperative PN after gastrectomy for gastric cancer. However, no randomized controlled trial has directly compared a high-protein PN formulation with a conventional formulation in adults undergoing curative gastric cancer surgery.

The PROGAIN trial was designed as a randomized, open-label, parallel-group superiority trial to evaluate whether perioperative administration of a high-protein PN formulation improves postoperative nitrogen balance compared with a conventional formulation in patients undergoing curative gastric cancer surgery. Nitrogen balance on postoperative day (POD) 5 was selected as the primary endpoint because it is an objective, quantifiable physiological marker of net protein metabolism that can be measured directly from 24-hour urinary urea nitrogen and dietary protein intake, has been used to evaluate perioperative protein supplementation in upper gastrointestinal cancer surgery [13,14,16], and is sensitive to short-term changes in protein delivery on a clinically relevant timescale. Although nitrogen balance has been used as a primary or principal endpoint in perioperative and nutritional randomized trials [17,18], an improvement is not guaranteed by increased protein delivery: in the catabolic state after major surgery, additional amino acids are frequently oxidized rather than retained, and several randomized trials of enriched amino-acid regimens have found no improvement in nitrogen balance [16,18]; the trial therefore tests whether this formulation produces genuine net nitrogen retention. We hypothesize that perioperative high-protein PN will improve nitrogen balance on POD 5, with secondary benefits on nutritional, glycemic, and functional recovery outcomes. The trial is registered at ClinicalTrials.gov (NCT07488611).

## Methods and analysis

### Study design and setting

PROGAIN is a single-center, prospective, parallel-group, two-arm, randomized, open-label superiority trial comparing high-protein PN with conventional PN in adults undergoing curative gastric cancer surgery, with 1:1 allocation between the investigational and control arms. The trial is conducted at the Department of Surgery, Soonchunhyang University Cheonan Hospital, Korea. The protocol is reported in accordance with the Standard Protocol Items: Recommendations for Interventional Trials (SPIRIT) 2025 statement [19] (the completed SPIRIT 2025 checklist is provided as S1 Checklist), and the trial will be reported at completion in accordance with the Consolidated Standards of Reporting Trials (CONSORT) 2025 statement [20]. The trial is registered at ClinicalTrials.gov (identifier NCT07488611).

### Eligibility criteria

Adults aged 19 years or older who are scheduled to undergo curative gastric cancer surgery and provide written informed consent are eligible. Detailed inclusion, exclusion and withdrawal criteria are presented in Table 1. Eligibility is based on the planned extent of resection: planned total and distal (subtotal) gastrectomy are eligible, whereas planned proximal gastrectomy is excluded; all standard reconstructions (Billroth I, Billroth II, Roux-en-Y) and surgical approaches (open, laparoscopic, robotic) are permitted. If the actual procedure differs from the plan (including an unplanned proximal gastrectomy), the participant is retained and analyzed in the allocated arm under the intention-to-treat principle, with the actual procedure recorded and examined in per-protocol and sensitivity analyses. Patients with clinically significant hepatic or renal impairment are excluded by the screening laboratory thresholds listed in Table 1, and hepatic and renal function are monitored daily during the intervention.

**Table 1:** Inclusion and exclusion criteria.

Inclusion criteria	Exclusion criteria
1. Adults ≥19 years of age scheduled to undergo curative gastric cancer surgery	1. Uncontrolled severe comorbidity, including: • Decompensated diabetes mellitus • Cerebrovascular or major cardiovascular event (acute myocardial infarction, stroke, pulmonary embolism) within the prior 6 months • Sepsis • Heart failure
2. Able and willing to provide written informed consent	2. Receipt of parenteral nutrition within 7 days prior to randomisation
	3. Severe preoperative metabolic abnormality on screening laboratory tests, defined as any of: • Triglyceride > 400 mg/dL • AST or ALT > 3 × upper limit of normal (ULN) • Total bilirubin > 3 × ULN • Serum creatinine > 2 × ULN • HbA1c > 9.0% • Potassium < 3.0 or > 6.0 mEq/L • Calcium > 12.5 mg/dL • Sodium > 155 mmol/L • Magnesium > 3.0 mg/dL
	4. Any other condition that, in the investigator’s judgment, makes the participant unsuitable for the trial 5. Planned proximal gastrectomy

Withdrawal criteria: participants who develop severe intra- or postoperative complications (e.g., massive hemorrhage requiring re-operation) or severe infusion-related events that preclude further peripheral parenteral nutrition (e.g., refractory infusion-site pain or phlebitis) will be withdrawn from the per-protocol analysis but retained in the intention-to-treat population.

### Recruitment

Potential participants are identified during preoperative outpatient consultations among patients scheduled for curative gastric cancer surgery. The enrolling investigator screens eligibility against the inclusion and exclusion criteria during the preoperative evaluation, provides verbal and written information about the trial — including its objectives, procedures, potential risks and benefits, and the participant’s right to withdraw at any time without consequence to standard of care — and obtains written informed consent before any trial-specific procedure is undertaken.

### Randomization and allocation concealment

**Random sequence generation.** Eligible participants are randomly allocated in a 1:1 ratio. The master allocation sequence was generated in advance for 120 sequential allocations using a custom Python script, with a fixed seed and permuted blocks of variable size (4 and 6) in randomly chosen order. Stratified randomization is not used because the anticipated low proportion of total gastrectomy would produce imbalanced strata and incomplete blocks in the small stratum; extent of gastric resection and baseline nutritional status are instead controlled analytically through a prespecified covariate-adjusted (ANCOVA) analysis, and a subgroup analysis by extent of resection (total versus distal gastrectomy) is prespecified as exploratory in the Statistical analysis section.

**Allocation concealment.** The master sequence is held in read-only form by a single dedicated clinical research coordinator (CRC) in a password-protected directory inaccessible to any other member of the trial team. The CRC has no role in determining participant eligibility, enrolling or consenting participants, administering the assigned interventions, or assessing study outcomes, ensuring that knowledge of the allocation sequence cannot influence enrollment decisions or outcome assessment. File integrity is verified by a SHA-256 cryptographic checksum computed at the time of sequence generation and stored separately, providing an audit trail against post-hoc modification; allocation concealment per se is ensured by the procedural safeguards described in the Allocation procedure section.

**Allocation procedure.** Once eligibility is confirmed and written informed consent obtained, the enrolling investigator notifies the CRC of the participant’s study identifier (PROGAIN-NNN, assigned in enrollment order). At each randomization request, the CRC opens the master sequence file in read-only mode, identifies and communicates only the next sequential assignment to the investigator, and does not preview upcoming allocations. The allocation is therefore disclosed to the enrolling investigator only after the participant’s eligibility has been confirmed and written informed consent obtained. The assignment is recorded immediately in a dedicated randomization log capturing study identifier, sequence number, group, date and time of allocation, allocator’s identifier, method, seed, and notes; the log is exported as a system-timestamped, non-editable PDF for archival after allocation updates or at predefined monitoring points*.*

**Blinding.** Because the products differ in labeling, blinding of participants, bedside investigators, and the ward nursing and nutrition support (NST) is not feasible; the trial is open-label for these parties. To minimize outcome-assessment bias, laboratory measurements (including the primary 24-hour urinary urea nitrogen assay), computed tomography–based skeletal muscle indices, and Clavien–Dindo classification of postoperative complications are performed without knowledge of group allocation. For the primary endpoint analysis, the dataset is prepared with intervention assignments replaced by coded group labels (Arm A/ Arm B), and daily amino-acid intake is recorded in grams without product identifiers. The analyst remains blinded to coded arm identity until the primary analysis is locked. Daily oral intake is recorded as a patient-reported outcome: after standardized in-hospital education, participants complete a meal-by-meal intake form that is verified by the nutrition support team; because this self-reported intake enters the numerator of the primary endpoint, it is a potential source of ascertainment bias in this open-label trial, which was one reason for selecting an objective, blinded assay (urinary urea nitrogen) for the primary endpoint. Postoperative complications are graded by an assessor blinded to allocation using the Clavien–Dindo classification, and standardized institutional discharge and diet-advancement criteria are applied. The coded group labels (Arm A/ Arm B) are unmasked by an independent individual only after database lock and finalization of the statistical analysis plan.

### Interventions

Investigational and control products. All participants will receive a once-daily three-in-one PN admixture corresponding to their assigned group. The investigational product is a high-protein (amino acid–enriched) peripheral PN formulation and the control product is a conventional peripheral PN formulation (Winuf A-plus peri and Winuf peri, respectively; JW Pharmaceutical, Seoul, Republic of Korea). Both products contain an identical lipid emulsion (a four-oil mixture of soybean oil, medium-chain triglycerides, olive oil, and fish oil at 30:25:25:20 by lipid weight, with an ω-6:ω-3 ratio of 2.1:1), so that the principal compositional difference between the two arms is the relative content of amino acids and dextrose. The investigational product delivers approximately 19% more amino-acid–derived nitrogen per millilitre and a correspondingly lower non-protein calorie-to-nitrogen (NPC/N) ratio than the control product (84 vs 109, respectively). Detailed compositional information is provided in Table 2. Because the two products differ simultaneously in amino-acid content, dextrose content, NPC/N ratio, electrolytes, osmolarity, and required infusion volume, the investigational arm represents a composite intervention (higher protein, lower carbohydrate, and lower NPC/N ratio); the expected daily delivered amounts of amino acids, nitrogen, glucose, and lipid for both arms at the scheduled coverage on each intervention day are provided in S4 Table, and this composite nature is considered in the interpretation of secondary outcomes.

**Table 2:** Composition of control and investigational peripheral parenteral nutrition products.

Component	Unit	Control (Winuf peri, conventional formulation)	Investigational (Winuf A-plus peri, amino acid–enriched)
**Composition per mL**			
Amino acids	mg/mL	31.5	37.4
Glucose (anhydrous)	mg/mL	70.7	64.4
Lipid	mg/mL	29.9	27.8
Nitrogen	mg/mL	5.21	6.17
Total energy	kcal/mL	0.69	0.67
**Energy and protein quality**			
Glucose: lipid energy ratio	%	50: 50	50: 50
Non-protein calorie-to-nitrogen ratio (NPC/N)	—	109	84
Essential amino acids/ total amino acids	%	45.3	45.3
Branched-chain amino acids/ total amino acids	%	19	19.1
**Lipid emulsion composition**			
Soybean oil	% of lipid	30	30
Medium-chain triglycerides	% of lipid	25	25
Olive oil	% of lipid	25	25
Fish oil	% of lipid	20	20
ω-6: ω-3 fatty acid ratio	—	2.1: 1	2.1: 1
**Electrolytes (per L)**			
Sodium (Na⁺)	mmol/L	25.3	29.9
Potassium (K⁺)	mmol/L	18.9	22.5
Magnesium (Mg²⁺)	mmol/L	3.16	3.75
Calcium (Ca²⁺)	mmol/L	1.59	1.88
Phosphate (PO_4_^3-^)	mmol/L	8.24	9.22
Zinc (Zn²⁺)	mmol/L	0.025	0.028
Chloride (Cl^-^)	mmol/L	34.6	41.1
Acetate (CH_3_COO^-^)	mmol/L	13	15.4
Sulfate (SO_4_²^-^)	mmol/L	3.18	3.77
**Physical properties**			
Osmolarity	mOsm/L	850	892
**Reference single-bag values (standard size)**			
Bag volume	mL	1085	1089
Amino acids	g	34.2	40.7
Glucose (anhydrous)	g	76.7	70.1
Lipid	g	32.4	30.3
Nitrogen	g	5.65	6.72
Total energy	kcal	751	730
Non-protein calories	kcal	614	567
**Manufacturer-stated maximum daily delivery (at the labelled maximum volume of 40 mL/kg/day)**			
Amino acids	g/kg/day	1.26	1.49
Glucose	g/kg/day	2.83	2.57
Lipid	g/kg/day	1.2	1.11
Total energy	kcal/kg/day	27.7	26.8
**Trial-protocol caloric target (back-calculated from 20 kcal/kg/day)**			
Resulting nitrogen delivery	g/kg/day	0.151	0.185
Required volume as % of manufacturer maximum (40 mL/kg/day)	%	73	75

Per-mL values are calculated from manufacturer specifications. Both products are three-in-one peripheral parenteral nutrition formulations manufactured by JW Pharmaceutical (Seoul, Republic of Korea). Bag sizes available at the participating institution: 1,085 mL and 2,020 mL for the control product, and 1,089 mL and 1,452 mL for the investigational product. The lipid emulsion composition is identical in the two products, isolating amino-acid and dextrose content as the principal compositional differences. The manufacturer-stated maximum daily volume of 40 mL/kg/day defines a regulatory ceiling rather than the trial-protocol dose. In the PROGAIN trial, the daily caloric target is back-calculated from a permissive hypocaloric target of 20 kcal/kg/day (using actual body weight when BMI < 25 and ideal body weight when BMI ≥ 25, with ideal body weight defined as height² × 23 in metres-squared and kilograms), and the required PN volume is delivered using a volumetric infusion pump. Abbreviations: BMI, body mass index; NPC/N, non-protein calorie-to-nitrogen ratio.

**Caloric target and dose calculation.** The daily caloric target during the intervention period is set at 20 kcal/kg/day, in keeping with current evidence supporting permissive hypocaloric feeding to avoid perioperative overfeeding. Body weight used for dose calculation is determined by body mass index (BMI): actual body weight is used for participants with BMI < 25 kg/m², and ideal body weight—calculated as height (m)² × 23 (a reference body-mass index of 23 kg/m², consistent with recommendations for Asian populations) [21], applied to both sexes—is used for those with BMI ≥ 25 kg/m². The required daily PN volume is back-calculated from the caloric target using the energy density of the assigned product (0.69 kcal/mL for the control and 0.67 kcal/mL for the investigational product), rounded to the nearest 1 mL, and delivered through a volumetric infusion pump. The calculated daily volume is not expected to exceed the manufacturer-stated maximum of 40 mL/kg/day. For a 75-kg participant, the control formulation delivers approximately 68 g of amino acid per day (0.9 g/kg) and the high-protein formulation approximately 84 g (1.1 g/kg); detailed examples are provided in S2 and S3 Tables. These amino-acid amounts represent PN-only delivery; the total protein supply (oral plus parenteral) that forms the basis of the nitrogen-balance calculation is higher, and the between-arm difference should be interpreted as a difference in PN-delivered rather than total protein.

**Stepwise PN delivery schedule.** PN is administered through a peripheral intravenous line; central venous access is not required. Peripheral access was chosen to avoid the infection and thrombosis risks of central venous catheterisation, consistent with the broader Enhanced Recovery After Surgery (ERAS) principle of minimizing invasive perioperative procedures. PN is initiated on the preoperative day (preoperative day −1; POD −1), withheld on the day of surgery (POD 0) because of perioperative anaesthetic and surgical considerations, and resumed from POD 1 through POD 5, for a total of six administration days. The PN dose on each day is delivered as a fixed proportion of the daily caloric target, decreasing stepwise as anticipated oral intake advances: 80% on POD −1, 100% on POD 1, 60% on POD 2 and POD 3, and 50% on POD 4 and POD 5. The stepwise schedule is summarized in Table 3.

**Table 3:** Stepwise perioperative parenteral nutrition delivery schedule.

Postoperative day	Oral diet stage	Anticipated mean oral energy intake (kcal/day)*	Scheduled PN coverage (% of daily caloric target)
POD −1	Preoperative oral carbohydrate loading	≈200 (per protocol)	80%
POD 0	Surgery — nil per os	—	PN withheld
POD 1	Sips of water	—	100%
POD 2	Post-gastrectomy liquid diet	≈350	60%
POD 3	Post-gastrectomy early soft diet	≈315	60%
POD 4–5	Post-gastrectomy soft diet	≈820	50%

PN, parenteral nutrition; POD, postoperative day. *Anticipated oral energy intake for POD 2 through POD 5 is derived from a 1-year retrospective audit of post-gastrectomy hospital meal intake at the participating institution (August 2024 – July 2025; n = 92, 160, and 102 measurements for the liquid, early soft, and soft diet stages, respectively); detailed values are provided in S1 Table. The daily caloric target is set at 20 kcal/kg/day, calculated using actual body weight when BMI < 25 kg/m² and ideal body weight (height [m]² × 23) when BMI ≥ 25 kg/m². The corresponding daily PN volume is back-calculated from the assigned product’s energy density (0.69 kcal/mL for the control and 0.67 kcal/mL for the investigational product), rounded to the nearest 1 mL, and delivered through a volumetric infusion pump. Preoperative oral carbohydrate loading is provided in two 200-mL servings on POD −1 of a commercially available carbohydrate-rich nutritional supplement (Nucare NoenPiO; Daesang Wellife, Republic of Korea), to maintain preoperative carbohydrate intake before the institutional mandatory fasting period (initiated from the evening of POD −1 owing to indocyanine green injection for sentinel lymph node mapping), in keeping with the underlying Enhanced Recovery After Surgery Society principle of avoiding prolonged preoperative fasting.

**Rationale for the stepwise schedule.** The proportional schedule was calibrated against an institutional 1-year retrospective audit (August 2024 – July 2025) of measured meal-by-meal oral intake yielding 354 stage-level intake measurements in post-gastrectomy inpatients at the participating center. Mean oral energy intake was approximately 350, 315, and 820 kcal/day for the post-gastrectomy liquid, early soft, and soft diet stages, respectively (S1 Table); the slightly lower energy at the early soft stage than the liquid stage reflects carbohydrate-drink supplementation of the liquid-diet stage and is also present in the standardized provided diet. Combined with PN coverage of 60%, 60%, and 50% of the daily caloric target, the schedule maintains total daily caloric delivery within approximately 80–120% of the 20 kcal/kg/day target across the body weight range typically encountered in elective gastrectomy (S2 Table), while reducing the risk of perioperative overfeeding.

**Concomitant nutritional care.** All participants follow the institutional standardized perioperative nutrition pathway, which is aligned with current ERAS Society recommendations [22] and is delivered by a multidisciplinary NST comprising a clinical pharmacist, a dietitian, a ward nurse, and an attending physician. In keeping with the institutional preoperative pathway, which requires fasting from the evening of POD −1 owing to indocyanine green injection for sentinel lymph node mapping, preoperative oral carbohydrate loading is administered on POD −1 in two 200-mL servings (approximately 200 kcal in total) of a commercially available carbohydrate-rich nutritional supplement to maintain adequate preoperative carbohydrate intake before the mandatory fasting period, in keeping with the underlying ERAS Society principle of avoiding prolonged preoperative fasting. Postoperatively, oral intake advances stepwise: sips of water from POD 1, a post-gastrectomy liquid diet on POD 2, a post-gastrectomy early soft diet on POD 3, and a post-gastrectomy soft diet on POD 4 and POD 5, advanced as tolerated. Daily oral intake is recorded meal by meal in 25% increments using a study-specific intake form (S1 File), from which oral energy and protein intake are calculated. The NST and the investigators may adjust supplementary fluids and electrolytes outside the assigned PN as clinically indicated; the assigned PN itself is not substituted with another commercial PN product during the intervention period. No oral nutritional supplements beyond the protocol-specified standardized perioperative diet are permitted during the intervention period; all oral intake, including the standardized supplement components of the diet, is recorded on the meal-by-meal intake form and included in the oral-protein term of the nitrogen-balance calculation.

**Monitoring and dose modification.** During the intervention period, blood glucose, serum electrolytes, hepatic and renal function tests, body weight, and urine output are monitored daily by the NST. The assigned PN may be temporarily withheld or reduced at the investigator’s discretion for predefined safety triggers, including blood glucose ≥250 mg/dL, clinically significant hepatic enzyme elevation, or mild to moderate infusion-site reactions. Permanent discontinuation of the allocated PN with withdrawal from the per-protocol population is reserved for severe intra- or postoperative complications — including anastomotic leak, bacteremia, or fungemia — and for severe infusion-related events that preclude further PN (e.g., refractory infusion-site pain or phlebitis); in such cases the allocated PN is discontinued at the discretion of the treating clinician, and the participant is retained in the intention-to-treat population and flagged for sensitivity analysis. Any modification or discontinuation of the assigned regimen, together with the reason, is documented on the case report form. Adherence to the allocated intervention is summarized as the proportion of planned PN doses received and the cumulative volume of allocated PN administered during POD −1 through POD 5.

### Outcomes

**Primary outcome.** The primary outcome is the nitrogen balance on POD 5, expressed in grams per day. Nitrogen balance is calculated using the standard simplified clinical formula: N balance (g/day) = [24-hour oral protein intake (g) ÷ 6.25 + 24-hour parenteral nitrogen delivered (g)] − (24-hour urinary urea nitrogen (g) + 4), where oral protein is converted using the divisor 6.25 (protein contains approximately 16% nitrogen by weight), parenteral nitrogen is derived directly from the product-specific nitrogen content (Table 2; 5.21 mg/mL for the control and 6.17 mg/mL for the investigational product) multiplied by the administered volume rather than from the amino-acid mass, and the constant 4 g accounts for non-urinary nitrogen losses through the integument and the gastrointestinal tract [23,24]. POD 5 was selected as the primary endpoint because previous prospective studies of perioperative protein supplementation in upper gastrointestinal cancer surgery have used assessment windows between POD 3 and POD 7, with POD 5 marking the transition from the catabolic phase that peaks within the first 48–72 hours after surgery to the early anabolic recovery phase [13,14,16]. The 24-hour urinary urea nitrogen collection is performed over POD 5 (00:00–24:00) by self-voided collection, since catheters are removed early under enhanced recovery; participants receive standardized instruction, and collection times and total urine volume are recorded as a completeness check, with incomplete collections flagged and handled per the missing-data plan (all participants remain hospitalized through POD 5). Participants with significant non-urinary gastrointestinal losses (high-output drainage, prolonged nasogastric aspiration, vomiting, or anastomotic leak) are flagged for sensitivity analysis. As limitations, the fixed 4 g constant does not capture gastrointestinal losses and completeness was not verified by creatinine; because both apply similarly across the randomized arms, residual error is expected to be non-differential.

**Secondary outcomes — nitrogen balance on POD 3.** To characterize the early trajectory of nitrogen metabolism, nitrogen balance is additionally assessed on POD 3 as a secondary outcome.

**Secondary outcomes — nutritional status.** Secondary nutritional outcomes include the skeletal muscle index measured at the third lumbar vertebral level on abdominopelvic computed tomography; composite nutritional and inflammatory–nutritional indices comprising the Nutritional Risk Screening 2002 (NRS-2002), GLIM criteria, Prognostic Nutritional Index (PNI), C-reactive protein–to-albumin ratio (CAR), and modified Glasgow Prognostic Score (mGPS); serum nutritional biomarkers (albumin, prealbumin, total protein); and longitudinal changes in body weight, body composition assessed by multifrequency segmental bioelectrical impedance analysis (InBody 770; InBody Co., Ltd., Seoul, Republic of Korea), and handgrip strength assessed by hand dynamometry.

**Secondary outcomes — glycemic control.** Postoperative glycemic outcomes comprise the proportion of participants with any blood glucose ≥180 mg/dL during POD 0 through POD 5, stratified by baseline diabetes status, together with the cumulative insulin requirement during the same period. Because the investigational formulation also delivers less carbohydrate, these glycemic outcomes partly reflect the compositional difference in carbohydrate load and are interpreted accordingly.

**Secondary outcomes — postoperative complications.** Postoperative complications occurring within 30 days after surgery are classified using the Clavien–Dindo classification. Specific complications captured include surgical site infection; pneumonia; delayed gastric emptying; and anastomotic leak.

**Secondary outcomes — functional recovery.** Functional recovery milestones aligned with the institutional ERAS pathway include time to first defecation, time to tolerate ≥50% of a soft diet for 24 hours, time to independent corridor ambulation, and length of postoperative hospital stay. Patient-reported recovery outcomes are assessed at POD 14, 1 month, 3 months, 6 months, and 12 months, and encompass oral intake (proportion relative to preoperative volume and meal frequency), early satiety, appetite loss, gastrointestinal symptoms (nausea, vomiting, dumping, reflux, diarrhoea, constipation), oral nutritional supplement use, and habitual physical activity. Clinical events—including emergency department visits, readmissions, and interventions—are captured through 12 months.

**Safety outcomes.** Adverse events, adverse drug reactions, and serious adverse events are recorded throughout the intervention and follow-up period, with investigator-assessed causality and severity. Predefined laboratory safety monitoring includes aspartate and alanine aminotransferase, blood urea nitrogen, serum creatinine, blood glucose, and serum electrolytes. Serious adverse events are reported within 24 hours to the manufacturer’s pharmacovigilance team in accordance with regulatory requirements. Infusion-site reactions are graded using the Visual Infusion Phlebitis score [25], and peripheral catheters are re-sited according to standard nursing practice; hyperglycemia (blood glucose ≥250 mg/dL) is managed with protocol-directed insulin. No formal data and safety monitoring board is convened, given the single-centre design, the short six-day intervention, and the use of two regulatory-approved PN products with established safety profiles; safety is overseen by the principal investigator with periodic institutional review board review.

**Fig 1:**
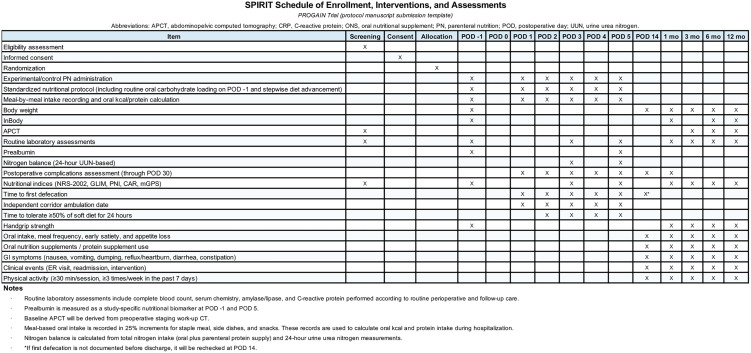
SPIRIT schedule of enrollment, interventions, and assessments for the PROGAIN trial. The figure displays the planned schedule of participant enrollment, intervention administration, and study assessments at each visit from screening through 12-month follow-up. *If first defecation is not documented before discharge, it will be rechecked at postoperative day 14. APCT, abdominopelvic computed tomography; CAR, C-reactive protein–to-albumin ratio; CRP, C-reactive protein; GLIM, Global Leadership Initiative on Malnutrition; mGPS, modified Glasgow Prognostic Score; NRS-2002, Nutritional Risk Screening 2002; ONS, oral nutritional supplement; PN, parenteral nutrition; PNI, Prognostic Nutritional Index; POD, postoperative day; UUN, urinary urea nitrogen.

Detailed measurement methods, timepoints, and prespecified statistical analyses for all primary and secondary outcomes are provided in Table 4. The full schedule of study assessments at each visit is summarized in Fig 1 (SPIRIT schedule).

**Table 4:** Primary, secondary, and safety outcomes with measurement methods, timepoints, and statistical analysis.

Outcome	Measurement/ definition	Timepoint(s)	Statistical analysis
**Primary outcome**			
Nitrogen balance on postoperative day 5	N balance (g/day) = [oral protein (g) ÷ 6.25 + parenteral nitrogen delivered (g)] − (urinary urea nitrogen (g) + 4); parenteral nitrogen is derived from the product-specific nitrogen content (Table 2) and administered volume [23,24]	POD 5	Two-sample t-test (or Wilcoxon rank-sum if non-normal); intention-to-treat as primary analysis
**Secondary outcomes — Nutritional status**			
Nitrogen balance trajectory	Same formula as the primary outcome, applied at the early postoperative time point	POD 3	Two-sample t-test or Wilcoxon rank-sum
Sarcopenia index	Skeletal muscle index measured at the third lumbar vertebral level on abdominopelvic computed tomography (cm²/m²)	Baseline, 3, 6, 12 months	Paired t-test (within-group); two-sample t-test (between-group); Chi-square or Fisher’s exact (sarcopenia incidence)
Nutritional risk and inflammatory–nutritional indices	Nutritional Risk Screening 2002 (NRS-2002), Global Leadership Initiative on Malnutrition (GLIM) criteria, Prognostic Nutritional Index (PNI), C-reactive protein–to-albumin ratio (CAR), modified Glasgow Prognostic Score (mGPS)	POD −1, POD 5, 1, 3, 6, 12 months	Two-sample t-test or Wilcoxon rank-sum (continuous); Chi-square or Fisher’s exact (categorical)
Serum nutritional biomarkers	Albumin, prealbumin, total protein	POD −1, POD 5; albumin/total protein additionally at 1, 3, 6, 12 months	Paired t-test (within-group); two-sample t-test (between-group)
Body weight, body composition, handgrip strength	Body weight; multifrequency segmental bioelectrical impedance analysis (InBody 770; InBody Co., Ltd., Seoul, Republic of Korea); handgrip dynamometry (kg)	Body weight: POD −1 and 14 days, 1, 3, 6, 12 months; InBody and handgrip: POD −1, 1, 6, 12 months	Linear mixed-effects model for repeated measures (random intercept per participant; fixed effects for treatment, time, and treatment × time interaction); between-group differences reported with 95% confidence intervals
**Secondary outcomes — Glycaemic control**			
Postoperative hyperglycaemia incidence	Proportion of participants with any blood glucose ≥180 mg/dL during POD 0–5, stratified by baseline diabetes status	POD 0–5	Chi-square or Fisher’s exact test; logistic regression adjusting for diabetes
Insulin requirement	Total insulin units administered during POD 0–5	POD 0–5	Two-sample t-test or Wilcoxon rank-sum test
**Secondary outcomes — Postoperative complications**			
Postoperative complications	All complications occurring through postoperative day 30, classified using the Clavien–Dindo classification; reported by grade and as the proportion at grade ≥II	Through POD 30	Chi-square or Fisher’s exact test
Specific complications	Surgical site infection; pneumonia; delayed gastric emptying; anastomotic leak	Through POD 30	Chi-square or Fisher’s exact test
**Secondary outcomes — Functional recovery**			
Time to first flatus/ defecation	First documented passage of flatus and stool	POD 1–14	Log-rank test; Kaplan–Meier estimates
Time to tolerate ≥50% of soft diet for 24 h	First day of meeting the threshold sustained for 24 h	POD 2–5	Log-rank test; Kaplan–Meier estimates
Time to independent corridor ambulation	First documented day of independent corridor walking	POD 1–5	Log-rank test; Kaplan–Meier estimates
Length of postoperative hospital stay	Days from surgery to discharge	Through discharge	Two-sample t-test or Wilcoxon rank-sum test
Patient-reported recovery (oral intake, GI symptoms, ONS use, activity)	Meal frequency, early satiety, appetite loss, nausea/vomiting, dumping, reflux, diarrhoea, constipation; oral nutritional supplement use; physical activity (≥30 min, ≥ 3 × /week)	POD 14, 1, 3, 6, 12 months	Two-sample t-test or Wilcoxon rank-sum (continuous); Chi-square or Fisher’s exact (categorical)
Clinical events	Emergency department visits, readmissions, interventions	Through 12 months	Chi-square or Fisher’s exact test; incidence rate ratio
**Safety outcomes**			
Adverse events, adverse drug reactions, and serious adverse events	Any adverse event, adverse drug reaction (per investigator’s causality assessment), or serious adverse event recorded throughout the intervention and follow-up period	Through 12 months	Descriptive (number of participants, events, incidence proportion); Chi-square or Fisher’s exact test for between-group comparison
Laboratory safety	Aspartate and alanine aminotransferase, blood urea nitrogen, serum creatinine, glucose, electrolytes	POD −1, POD 3, POD 5, 1, 3, 6, 12 months	Descriptive summary; between-group comparison at each timepoint

All analyses will be performed on the intention-to-treat population, with per-protocol analysis as a sensitivity analysis. Two-sided α = 0.05 will be used throughout. The single primary endpoint is the sole confirmatory analysis. The two key secondary endpoints — POD 3 nitrogen balance (reported with the nitrogen balance trajectory) and the early change in serum prealbumin, defined as the change from the preoperative (POD −1) baseline to POD 5 — are controlled for family-wise error at 0.05 by the Holm procedure, independently of the primary result; all other secondary outcomes are exploratory and reported with 95% confidence intervals without multiplicity adjustment. For the primary endpoint, genuine missing data are handled by multiple imputation under the missing-at-random assumption as the primary approach, with complete-case analysis as a sensitivity analysis and a delta-adjusted (tipping-point) analysis under missing-not-at-random assumptions. Full specifications are given in the dated statistical analysis plan (SAP v1.0, 21 July 2026). Abbreviations: APCT, abdominopelvic computed tomography; CAR, C-reactive protein–to-albumin ratio; GLIM, Global Leadership Initiative on Malnutrition; mGPS, modified Glasgow Prognostic Score; NRS-2002, Nutritional Risk Screening 2002; ONS, oral nutritional supplement; PNI, Prognostic Nutritional Index; POD, postoperative day.

### Sample size

The primary outcome is the nitrogen balance on POD 5, expressed in grams per day, compared between the two arms. The sample size calculation is based on a two-sample t-test of independent means. In the absence of a directly comparable randomized trial of high-protein versus conventional PN in patients undergoing gastric cancer surgery, the assumed effect size and variability were derived from related studies of postoperative nitrogen balance under modified protein and amino-acid support [13,14] and from clinical considerations of the smallest between-group difference that would be regarded as clinically meaningful in this population. A between-group mean difference of 3 g/day in postoperative nitrogen balance was assumed, with a common standard deviation of 5 g/day. Using a two-sided significance level of 0.05 and statistical power of 80%, the minimum required sample size is 44 participants per arm (88 participants in total). Anticipating a combined withdrawal, protocol non-completion, or missing primary outcome rate of approximately 20%, the planned enrollment is increased to 55 participants per arm, giving a total target enrollment of 110 participants. The 20% inflation is an allowance for missingness of the POD 5 primary outcome — incomplete 24-hour collection, early events, PN discontinuation, or death before POD 5 — rather than attrition during the 12-month follow-up; it is a conservative estimate consistent with the completeness typically achievable for a fixed-day metabolic endpoint and with attrition reported in comparable post-gastrectomy nutrition trials [26,27]. Because scheduled PN coverage on POD 5 is 50%, the between-arm difference in delivered nitrogen on the primary-endpoint day is approximately 1.0–1.2 g/day, with a cumulative difference of roughly 8 g over the six-day intervention; the assumed 3 g/day balance difference therefore exceeds the single-day intake difference and, if observed, would indicate a genuine difference in nitrogen retention rather than intake alone. The sensitivity of statistical power to the assumed effect size and standard deviation is summarized in S5 Table.

### Data collection and management

Trial data are recorded by the enrolling investigator and the CRC on a study-specific case report form. Source documents include the participant’s electronic medical record, laboratory results, abdominopelvic computed tomography images, and the participant-completed meal-by-meal oral intake form; each entry is dated and initialled by the recorder, and entries are reviewed for completeness and consistency by the CRC with discrepancies resolved against source documents. Trial documents identifying participants are stored in locked, access-controlled facilities at the participating institution; participants are identified in trial datasets by a study identifier (PROGAIN-NNN) without direct personal identifiers. Trial records will be retained in accordance with applicable Korean regulations and institutional requirements.

### Statistical analysis

**Analysis populations.** All efficacy analyses are performed in the intention-to-treat (ITT) population, comprising all randomized participants analyzed in the group to which they were allocated. The per-protocol population is defined as participants who completed the planned PN intervention through POD 5, received at least 90% of the planned cumulative allocated PN volume or amino acid dose, completed the POD 5 nitrogen balance assessment, and had no prespecified major protocol deviations; this population will be used for sensitivity analyses of the primary and key secondary outcomes. The safety population comprises all participants who received at least one administration of allocated PN.

**Primary analysis.** The primary outcome is compared between the investigational and control arms using a two-sample t-test, with the between-group mean difference reported with a 95% confidence interval; the Wilcoxon rank-sum test is used as a sensitivity analysis if distributional assumptions are clearly violated. In a prespecified adjusted analysis, POD 5 nitrogen balance is modelled by analysis of covariance (ANCOVA) with treatment arm as the factor and extent of gastric resection and baseline nutritional status as covariates.

**Mechanistic analyses of the primary endpoint.** To separate the arithmetic and metabolic components of the endpoint, prespecified mechanistic analyses compare between arms (a) total nitrogen intake, (b) urinary urea nitrogen excretion, and (c) net nitrogen retention on POD 5, and evaluate nitrogen-utilization efficiency (net retention relative to delivered nitrogen), and, in an exploratory analysis, adjust the primary endpoint for delivered nitrogen as a covariate. Because delivered nitrogen is a post-randomization variable largely determined by treatment allocation, adjustment for it cannot by itself causally separate nitrogen delivery from nitrogen utilization; these analyses are exploratory and descriptive, use already-recorded variables, and are reported with 95% confidence intervals as hypothesis-generating without causal claims. The partial dependence of nitrogen balance on nitrogen intake is acknowledged as a limitation that these analyses are designed to characterize.

**Secondary outcomes, subgroups, and missing data.** The single primary endpoint is the sole confirmatory analysis (two-sided α = 0.05). Two key secondary endpoints — POD 3 nitrogen balance and early change in prealbumin, both objective, blinded, and responsive to short-term protein delivery — are controlled for family-wise error at 0.05 by the Holm procedure, independently of the primary result. All other outcomes are exploratory, reported as point estimates with 95% confidence intervals without multiplicity adjustment and interpreted as hypothesis-generating; postoperative complications are additionally analyzed with multivariable adjustment for extent of resection, surgical approach, and comorbidity, and a subgroup analysis by extent of resection (total versus distal gastrectomy) is prespecified as exploratory via a treatment-by-subgroup interaction. Following ICH E9(R1), the primary estimand is the between-arm difference in mean POD 5 nitrogen balance in the intention-to-treat population under a treatment-policy strategy: PN dose reduction or discontinuation is handled by using the observed POD 5 value and is not treated as missing. Genuine missing data (death before POD 5 or failed collection) are addressed by multiple imputation under missing-at-random as the primary approach, with complete-case analysis as sensitivity; because complication-related missingness may be non-random, a delta-adjusted (tipping-point) sensitivity analysis is performed.

**Other considerations.** No interim efficacy analysis is planned. The full statistical analysis plan, including detailed analysis specifications for each outcome, is finalised and dated before release of the coded treatment labels for the primary analysis. All analyses are conducted using R (R Foundation for Statistical Computing, Vienna, Austria).

### Safety monitoring

Adverse events, adverse drug reactions, and serious adverse events are identified throughout the intervention and follow-up period through systematic review by the NST — comprising a clinical pharmacist, a dietitian, a ward nurse, and an attending physician — supplemented by direct enquiry, clinical examination, laboratory monitoring, and review of medical records. Events are graded for severity and assessed for causality by the investigator. Serious adverse events are reported within 24 hours to the pharmacovigilance department of the manufacturer of the investigational and control products and to the institutional review board (IRB), in accordance with applicable Korean regulations. Predefined safety triggers for withholding or reducing the assigned PN are described in the Interventions section. No formal data and safety monitoring board is convened given the single-center design, the short intervention period, and the use of two regulatory-approved PN products with established safety profiles; trial safety is overseen by the principal investigator with periodic review by the IRB.

### Patient and public involvement

**Fig 2:**
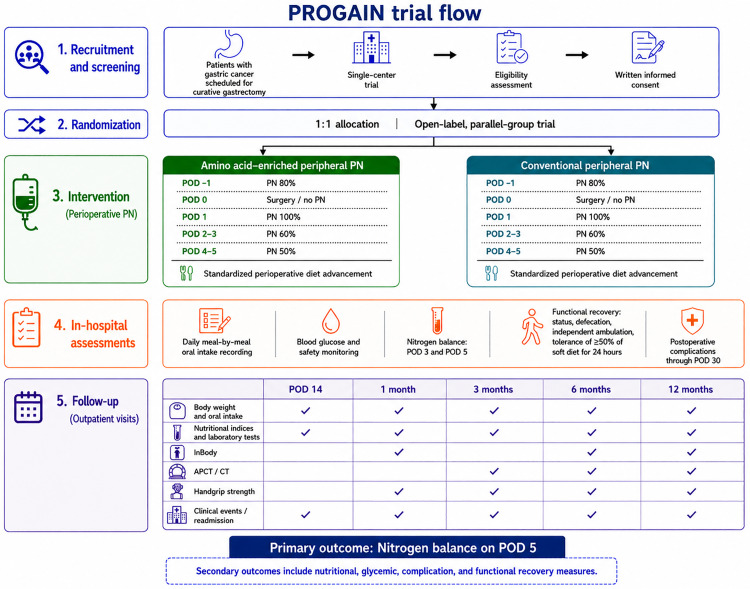
PROGAIN trial flow from recruitment through follow-up. Schematic overview of the trial conduct. Eligible patients are screened, randomized 1:1 to amino acid–enriched (high-protein) peripheral PN or conventional peripheral PN in an open-label, parallel-group design, and receive the assigned PN from preoperative day −1 through postoperative day (POD) 5 alongside standardized perioperative diet advancement. Daily intervention-period assessments include meal intake logs, with blood glucose monitoring on POD 5 and skeletal muscle index assessed from baseline and follow-up computed tomography, and laboratory and functional recovery measures. Outpatient follow-up visits at POD 14 and at 1, 3, 6, and 12 months capture body weight, nutritional indices, mGPS, CAR/PNI, and clinical events. The primary outcome is nitrogen balance on POD 5; secondary outcomes include nutritional, glycemic, complication, and functional recovery measures. CAR, C-reactive protein–to-albumin ratio; mGPS, modified Glasgow Prognostic Score; PN, parenteral nutrition; PNI, Prognostic Nutritional Index; POD, postoperative day.

Patients and members of the public were not involved in the design, conduct, or reporting of this research. Aggregate trial results will be communicated to participants through their treating physician at the participating institution upon completion of the trial. A schematic overview of the trial conduct, integrating recruitment, randomization, intervention administration, in-hospital assessments, and outpatient follow-up, is provided in Fig 2.

### Ethics and dissemination

The trial is conducted in accordance with the Declaration of Helsinki, the International Council for Harmonisation Good Clinical Practice guidelines, and applicable Korean regulations. The protocol, the participant information sheet, and the informed consent form have been approved by the IRB of Soonchunhyang University Cheonan Hospital (approval number: 2025-11-024-001); The full IRB-approved study protocol (most recent amended version) is provided as S1 Protocol. IRB approval is obtained for any subsequent protocol amendment before its implementation, and substantial amendments are notified to the trial registry. Written informed consent is obtained from each participant prior to any trial-specific procedure, as described in the Recruitment section.

Trial findings will be disseminated through publication in a peer-reviewed scientific journal in accordance with the CONSORT 2025 statement (an anticipated participant flow diagram prepared from the CONSORT 2025 template is provided as S1 Fig and will be populated at trial completion), through presentations at scientific meetings, and through results reporting on ClinicalTrials.gov upon trial completion. De-identified individual participant data underlying the published results will be made available, on reasonable request to the corresponding author and following review by the institutional data access committee and applicable ethical and regulatory requirements*.*

### Trial status

At the time of submission, the trial is actively recruiting. The trial received IRB approval on 5 December 2025, the first participant was randomized on 13 February 2026, and the trial was registered at ClinicalTrials.gov (NCT07488611) on 14 March 2026, approximately 29 days after first enrollment. Recruitment is expected to be completed in February 2027; the anticipated primary completion date (last collection of the primary outcome, postoperative day 5 nitrogen balance) is February 2027, and the anticipated study completion date (last participant follow-up assessment) is March 2028, consistent with the trial registry record. Primary and secondary outcome results are anticipated to be reported by late 2028 to mid-2029, following completion of data cleaning, monitoring, and statistical analysis. The current protocol version is 1.2 (approved 16 July 2026), and the present manuscript reflects this version. We acknowledge that public registration was completed after first participant enrollment owing to administrative processing of access to the institutional registration system. The IRB-approved protocol and the sponsor agreement were finalized before the first participant was enrolled, and the registry record corresponds fully to the IRB-approved protocol, with no change to eligibility, the intervention, the primary or secondary outcomes, or the statistical analysis plan. By the registration date, four participants had passed POD 5 by calendar date; a confirmed POD 5 primary-endpoint result was available for three of them (the fourth, operated on 6 March 2026, did not yet have a confirmed result, as 14 March was a weekend). Because the outcomes and analysis plan had been fixed in the IRB-approved protocol before enrollment, the availability of these early results did not permit any post hoc selection or modification of outcomes or analyses. The trial has used non-stratified block randomization consistently from the first participant; all participants randomized to date were allocated by this single, unchanged method. To align the protocol document with the trial as conducted, the description of randomization was revised from stratified to non-stratified block randomization with prespecified covariate adjustment and approved by the IRB as protocol amendment version 1.2 on 16 July 2026 (SCHCA IRB 2025-11-024-003). This amendment corrects the protocol document rather than the conduct and affects neither eligibility, the intervention, the endpoints, nor the target sample size. The prespecified analyses (covariate-adjusted ANCOVA, exploratory mechanistic nitrogen analyses, multiplicity procedure, and missing-data strategy) are documented in a dated statistical analysis plan (SAP v1.0, 21 July 2026), finalized before any aggregate or between-group analysis of trial data. The authors confirm that all ongoing and related trials for this drug/intervention are registered. All future related trials will be registered prospectively.

## Supporting information

S1 Fig CONSORT 2025 participant flow diagram template for the PROGAIN trial. Anticipated participant flow through enrollment, allocation, follow-up, and analysis prepared according to the CONSORT 2025 statement; the diagram will be populated with actual participant counts at trial completion.
(TIF)

S1 Table Institutional retrospective audit of meal-by-meal oral intake (354 stage-level measurements) in post-gastrectomy inpatients (August 2024 – July 2025). Anticipated postoperative oral energy intake by diet stage used to calibrate the stepwise PN schedule.
(XLSX)

S2 Table Worked examples of caloric and amino acid delivery for the control and investigational PN products at representative body weights, illustrating the relationship between assigned PN volume and total daily caloric/protein delivery. (XLSX)

S3 Table Comparison of cumulative protein delivery between the control and investigational arms across the stepwise PN schedule at 60–100% PN coverage of the 20 kcal/kg/day target. (XLSX)

S4 Table Expected daily delivered amounts of amino acids, nitrogen, glucose, and lipid (with total volume) for the control and investigational arms at the scheduled PN coverage on each intervention day (preoperative day −1 through POD 5). (XLSX)

S5 Table Sensitivity of statistical power for the primary endpoint to the assumed between-arm difference (2.0, 2.5, 3.0 g/day) and standard deviation (4, 5, 6 g/day), at n = 44 evaluable participants per arm and two-sided α = 0.05. (XLSX)

S1 File Participant-completed meal-by-meal oral intake form used to record postoperative oral energy and protein intake at each meal during the intervention period. (DOCX)

S1 Protocol The full IRB-approved study protocol (most recent amended version) submitted to and approved by the Institutional Review Board of Soonchunhyang University Cheonan Hospital. (DOCX)

S1 Checklist Completed SPIRIT 2025 reporting checklist for the PROGAIN study protocol manuscript. (XLSX)
