# Supplementary material for: The PROGAIN trial: A randomized controlled trial of high-protein peripheral parenteral nutrition on nitrogen balance and recovery after gastric cancer surgery — study protocol
Source: PLoS One. 2026 Aug 7;21(8):e0355677. doi: 10.1371/journal.pone.0355677 (PMC13450730; doi:10.1371/journal.pone.0355677)
Supplement: Supplementary file 1 [file pone.0355677.s007.docx]

**Oral Food Intake Assessment Form**

| **Oral Food Intake Record Form for Hospital Meals** | | | | | | |
| --- | --- | --- | --- | --- | --- | --- |
| **Day** | **Meal** | **Component** | **0–25%** | **25–50%** | **50–75%** | **75–100%** |
| **POD #1 (Pre-op day –1)** | Preoperative carbohydrate drink  (Newcare NO-NPO) × 2 | | ☐ | ☐ | ☐ | ☐ |
| **POD #2**  **Post-gastrectomy liquid diet** | **Breakfast** | Rice gruel 1/2 bowl | ☐ | ☐ | ☐ | ☐ |
|  |  | Rice gruel 1/2 bowl | ☐ | ☐ | ☐ | ☐ |
|  |  | Carbohydrate drink × 1 | ☐ | ☐ | ☐ | ☐ |
|  | **Lunch** | Rice gruel 1/2 bowl | ☐ | ☐ | ☐ | ☐ |
|  |  | Rice gruel 1/2 bowl | ☐ | ☐ | ☐ | ☐ |
|  |  | Carbohydrate drink × 1 | ☐ | ☐ | ☐ | ☐ |
|  | **Dinner** | Rice gruel 1/2 bowl | ☐ | ☐ | ☐ | ☐ |
|  |  | Rice gruel 1/2 bowl | ☐ | ☐ | ☐ | ☐ |
|  |  | Carbohydrate drink × 1 | ☐ | ☐ | ☐ | ☐ |
| **POD #3**  **post-gastrectomy early soft diet** | **Breakfast** | Thick rice porridge | ☐ | ☐ | ☐ | ☐ |
|  |  | Soft tofu | ☐ | ☐ | ☐ | ☐ |
|  | **Lunch** | Thick rice porridge | ☐ | ☐ | ☐ | ☐ |
|  |  | Steamed egg | ☐ | ☐ | ☐ | ☐ |
|  |  | Plain yogurt | ☐ | ☐ | ☐ | ☐ |
|  | **Dinner** | Thick rice porridge | ☐ | ☐ | ☐ | ☐ |
|  |  | Soft tofu | ☐ | ☐ | ☐ | ☐ |
| **POD #4**  **Post-gastrectomy soft diet** | **Breakfast** | Rice porridge | ☐ | ☐ | ☐ | ☐ |
|  |  | Soup + side dishes | ☐ | ☐ | ☐ | ☐ |
|  |  | Plain yogurt | ☐ | ☐ | ☐ | ☐ |
|  | **Lunch** | Rice porridge | ☐ | ☐ | ☐ | ☐ |
|  |  | Soup + side dishes | ☐ | ☐ | ☐ | ☐ |
|  |  | High-protein jelly | ☐ | ☐ | ☐ | ☐ |
|  | **Dinner** | Rice porridge | ☐ | ☐ | ☐ | ☐ |
|  |  | Soup + side dishes | ☐ | ☐ | ☐ | ☐ |
|  |  | Low-sugar soy milk | ☐ | ☐ | ☐ | ☐ |
| **POD #5**  **Soft diet post-gastrectomy** | **Breakfast** | Rice porridge | ☐ | ☐ | ☐ | ☐ |
|  |  | Soup + side dishes | ☐ | ☐ | ☐ | ☐ |
|  |  | Plain yogurt | ☐ | ☐ | ☐ | ☐ |
|  | **Lunch** | Rice porridge | ☐ | ☐ | ☐ | ☐ |
|  |  | Soup + side dishes | ☐ | ☐ | ☐ | ☐ |
|  |  | High-protein jelly | ☐ | ☐ | ☐ | ☐ |
|  | **Dinner** | Rice porridge | ☐ | ☐ | ☐ | ☐ |
|  |  | Soup + side dishes | ☐ | ☐ | ☐ | ☐ |
|  |  | Low-sugar soy milk | ☐ | ☐ | ☐ | ☐ |
