# Supplementary material for: The PROGAIN trial: A randomized controlled trial of high-protein peripheral parenteral nutrition on nitrogen balance and recovery after gastric cancer surgery — study protocol
Source: PLoS One. 2026 Aug 7;21(8):e0355677. doi: 10.1371/journal.pone.0355677 (PMC13450730; doi:10.1371/journal.pone.0355677)
Supplement: Supplementary file 2 [file pone.0355677.s008.docx]

**영문 제목: The PROGAIN Trial:
*A randomized study of protein-enriched parenteral nutrition on nitrogen balance and recovery in perioperative gastric cancer surgery*
*(PROtein-enriched Parenteral nutrition in GAstric cancer: clinical INvestigation)***

**연구자 주도 임상 시험**

***고아미노산 영양 수액이 위암 수술 환자의 영양 상태 및 수술 후 회복에 미치는 효과:***

***전향적, 단일기관, 무작위배정(Randomized Controlled Trial) 중재연구***

***책임연구자 순천향대학교 천안병원 송금종***

***2026.07.06***

***ver.1.2***

**목차**

[1.	일반 정보	4](#_Toc3449665)

[1.1	임상시험 제목	4](#_Toc3449666)

[1.2	임상시험 실시기관 및 책임연구자	4](#_Toc3449667)

[1.2.1	실시 기관	4](#_Toc3449668)

[1.2.2	책임연구자 및 소속, 연락처	4](#_Toc3449669)

[1.3	공동연구자 및 소속 *(필요시)*	4](#_Toc3449670)

[1.4	연구개요	5](#_Toc3449671)

[2.	임상시험의 배경 및 필요성	6](#_Toc3449672)

[3.	임상시험의 목적	6](#_Toc3449673)

[4.	임상시험을 통하여 예측되는 결과	6](#_Toc3449674)

[5.	임상시험용 의약품의 정보	6](#_Toc3449675)

[5.1	시험약	6](#_Toc3449676)

[5.2	대조약	6](#_Toc3449677)

[6.	임상시험 기간	6](#_Toc3449678)

[7.	시험대상자	7](#_Toc3449679)

[7.1	선정기준	7](#_Toc3449680)

[7.2	제외기준	7](#_Toc3449681)

[7.3	대상자 수 및 산출 근거	7](#_Toc3449682)

[7.3.1	대상자 수	7](#_Toc3449683)

[7.3.2	산출 근거	7](#_Toc3449684)

[8.	연구 방법	9](#_Toc3449685)

[8.1	일정표	9](#_Toc3449686)

[8.2	치료 방법 및 투약 계획	10](#_Toc3449687)

[8.3	검사항목 및 목적	10](#_Toc3449688)

[9.	평가 항목	11](#_Toc3449689)

[9.1	일차 유효성 평가변수	11](#_Toc3449690)

[9.2	이차 유효성 평가변수	11](#_Toc3449691)

[9.3	안전성 평가변수	11](#_Toc3449692)

[9.4	기타 평가변수	11](#_Toc3449693)

[10.	통계 분석 방법	11](#_Toc3449694)

[10.1	일차 유효성 평가	11](#_Toc3449695)

[10.2	이차 유효성 평가	12](#_Toc3449696)

[10.3	안전성 평가	12](#_Toc3449697)

[11.	참고문헌	12](#_Toc3449698)

***※(필요시) 항목 제외하고는 모두 기재, 목차 필드 업데이트하여 제출***

# 1. 일반 정보

## 1.1 임상시험 제목

고아미노산 영양 수액이 위암 수술 환자의 영양 상태 및 수술 후 회복에 미치는 효과: 전향적, 단일기관, 무작위배정(Randomized Controlled Trial) 중재연구

임상시험 실시기관 및 책임연구자

### 1.1.1 실시 기관

총 1개 기관

순천향대학교 천안병원

### 1.1.2 책임연구자 및 소속, 연락처

순천향대학교 천안병원 부교수 송금종

## 1.2 공동연구자 및 소속 *(필요시)*

순천향대학교 천안병원 교수 손명원

순천향대학교 천안병원 조교수 윤종혁

순천향대학교 천안병원 임상강사 신현섭

순천향대학교 천안병원 류태의 연구원

## 1.3 연구개요

**1) 임상시험 제목 및 디자인**

고아미노산 영양 수액이 위암 수술 환자의 영양 상태 및 수술 후 회복에 미치는 효과: 전향적, 단일기관, 무작위배정(Randomized Controlled Trial) 중재연구

2) 임상시험 기간

IRB승인일 ~ 3년

1) 연구 준비 기간: IRB 승인 및 계약 준비 기간

2) 대상자 모집 기간: IRB 승인일로부터 1년 6개월

3) 대상자 관찰 기간: 수술일로부터 12개월

4) 데이터 분석 및 결과 보고서 작성 기간: 대상자 관찰 기간 종료로부터 6개월

3) 임상시험의 필요성 요약

영양 불량은 위암 환자에서 흔하며, 항암 치료 순응도, 수술 후 합병증, 생존율과 밀접한 관련이 있다. 수술 후 회복기에는 충분한 단백질 및 열량 공급이 필수적이나, 위절제술 이후에는 경구 섭취가 제한되는 경우가 많아 비경구영양(TPN)의 역할이 중요하다. 고아미노산 TPN(4세대, 위너프에이플러스)은 기존 제제보다 효율적인 단백질 공급이 가능하다는 점에서 수술 후 회복에 유리할 수 있으며, 이에 대한 근거를 마련하기 위한 연구가 필요하다.

**4) 임상시험용 의약품 또는 투여되는 약물 정보 *(필요 시)***

시험약: 위너프에이플러스 주 (고아미노산 함유, 4세대 TPN, 1500ml/1000ml peripheral)

대조약: 위너프 주 (기존 TPN, peripheral)

**6) 시험대상자**

- 대상질환: 위암
- 목표 대상자 수: 110명

**7) 임상시험 방법**

연구 대상자는 수술 전 외래 또는 입원 중 대상자 적합성 선별을 거쳐 연구 설명 및 동의서를 취득한 후 등록된다. 등록된 대상자는 예정된 수술 범위(전절제 vs 부분절제)에 따라 층화되며, 각 층 내에서 블록 무작위 배정표에 따라 시험군 또는 대조군에 1:1로 배정된다. 배정은 등록 직후 시행되며, 배정표는 사전 생성된 엑셀 형식의 순차표를 이용한다.

시험군과 대조군은 모두 수술 전 1일(POD -1)과 수술 후 1-5일에 걸쳐 총 6일간 TPN을 투여받는다. 모든 대상자는 **총 일일 수액 요구량 중 최소 1,500mL 이상을 정맥영양수액으로 공급받는다.** 수액은 말초 정맥을 통해 주입되며, 부작용 여부 및 생화학적 수치 모니터링이 병행된다.

POD 5일차에 일차 유효성 평가변수인 질소 균형(Nitrogen balance)을 측정하며, 퇴원 시점과 수술 후 1개월, 3개월, 6개월, 최대 12개월 시점까지 이차 유효성 평가변수(CT 기반 근육량 변화, 영양 지표, 기능 회복 등)에 대한 추적관찰이 진행된다. 수술 후 혈당 조절 상태, 감염성 합병증(DGE 등) 여부 또한 이차 유효성 평가 변수로 측정한다.

수액 관련 이상반응 등은 안전성 평가변수로 기록되며, 모든 이상사례는 정의된 기준에 따라 문서화 및 보고된다.

필요 시 대상자의 실제 수술 범위가 계획과 다르더라도 배정 당시 군을 기준으로 분석을 진행하는 **Intention-to-Treat (ITT)** 원칙을 적용한다.

**8) 평가항목**

- 일차 유효성 평가변수: 수술 후 5일째(Postoperative Day 5)의 질소 균형(Nitrogen balance, g/day)
- 이차 유효성 평가변수: 수술 후 최대 12개월까지의 중장기 영양 지표 개선 효과-

Nutritional index(Sarcopenia, CRP-Albumin ratio, mGPS, NRI, PNI 등), 수술 후 혈당 변화, 수술 후 합병증 발생률, 기능적 회복 지표(경구 섭취량, 입원 기간, 보행 시점 및 통증 강도 등)

- 안전성 평가변수: 이상사례(Adverse Event), 약물이상반응(Adverse Drug Reaction), 중대한 이상사례(Serious AE)

# 2. 임상시험의 배경 및 필요성

위암은 전 세계적으로 높은 발병률과 사망률을 보이는 질환이며, 특히 아시아 국가, 그 중에서도 한국에서의 유병률은 상대적으로 높다. 수술은 국소 진행성 위암의 근치적 치료법의 핵심이나, 위절제술은 영양소 섭취와 대사에 중대한 영향을 미치며, 수술 전후로 심각한 영양 불균형 및 질소 손실이 빈번하게 발생한다(1).

암 환자에서 영양불량(malnutrition)은 30.9%에서 최대 83%까지 다양한 빈도로 보고되며, 이는 암의 위치와 연령에 따라 상이하다. 특히 암 관련 근감소(cachexia, sarcopenia)는 흔히 동반되며, 암 종류에 따라 근감소증이 20–70%, 악액질이 50–80%의 환자에서 발생하는 것으로 알려져 있다.(2-4) 특히 위절제술을 받은 환자에서는 경구 섭취 감소, 흡수 불량, 염증 반응의 지속 등으로 인해 음성 질소 균형(negative nitrogen balance)이 발생하고, 이는 단백질 손실과 근육량 감소로 이어져 수술 후 회복 지연, 감염 위험 증가, 항암치료 순응도 저하 등 부정적인 결과를 초래한다(5, 6). 또한 최근 근육량 감소와 연관된 GLIM criteria 영양 지표가 위암 수술 후 환자에서 항암 치료 순응도 저하 및 생존율 저하로 이어진다는 것이 밝혀졌다.(7)

이러한 배경에서 수술 전후 환자에게 적절한 정맥영양요법(TPN)을 시행하는 것은 생리적 질소 균형을 유지하고, 영양 상태를 개선하여 임상 예후를 향상시킬 수 있는 중요한 중재로 간주된다. 특히 고단백 아미노산 비율을 높인 4세대 TPN 제제(위너프에이플러스주)는 기존 제제보다 단백질 합성에 유리한 조성비(고단백, 저포도당)로 설계되어 있어, 수술 후 질소 손실을 보다 효과적으로 보완할 수 있을 것으로 기대된다. 특히, 이론적으로 고아미노산 영양 수액을 통한 수술 전후 영양 개입을 통해 위암 수술 후 환자의 단백 손실과 근육량 감소를 줄이는데 기여할 수 있을 것으로 보인다.

국제영양학회(ESPEN) 가이드라인은 암 환자에서 단백질 섭취를 최소 1.0 g/kg/day 이상, 필요 시 1.2–2.0 g/kg/day까지 권고하고 있으며, 이는 건강한 성인의 기준치(0.8 g/kg/day)보다 높다.(8, 9) 특히 고령 환자나 중등도 이상의 영양불량 환자에서는 2.0 g/kg/day까지의 고단백 보충이 근육량 유지 및 회복에 필요할 수 있으며(10), 고단백 정맥영양(>1.5 g/kg/day)의 유효성은 중환자 대상 연구에서 이미 입증된 바 있다(11).

따라서, 수술 전후 고단백 TPN이 위암 환자의 근육 손실을 예방하고 영양 상태 및 수술 후 회복을 개선할 수 있다는 가설은 임상적으로 충분한 근거와 타당성을 가진다.

그러나, 현재까지 위절제술을 받은 위암 환자에서 4세대 고단백 정맥영양수액제의 임상적 효과(질소 균형, 영양지표, 기능 회복 등)를 전향적으로 평가한 무작위배정 임상시험은 전무한 실정이며, 실제 임상에서는 적절한 제제 선택 기준이 마련되어 있지 않아 일관된 치료 전략 수립이 어려운 상황이다.(12)

이에 본 연구는 위암 수술 후 정맥영양요법의 표준화 및 고단백 TPN 제제의 임상적 유용성을 검증하고자 한다. 생리학적으로 명확하며 고단백 영양의 효과를 측정함에 있어 흔히 사용되는 질소 균형(nitrogen balance)를 primary endpoint를 설정하여 위너프에이플러스주(4세대 고단백 TPN)의 효과를 평가하고자 하며, 이는 향후 수술 후 영양 치료 전략 수립에 중요한 근거 자료로 활용될 수 있을 것이다(13, 14).

# 3. 임상시험의 목적

본 연구는 위암 수술 환자에서 고단백 영양 수액의 수술 후 영양 상태 및 회복에 미치는 효과를 평가하기 위한 무작위배정 전향적 단일기관 임상시험이다.

주요 목적은 고단백 영양 수액 투여군과 기존 3세대 TPN 투여군 간의 수술 후 영양 균형 및 회복 양상의 차이를 비교하고, 고단백 정맥영양요법의 임상적 유용성을 입증하는 것이다.

주요 및 부차적 평가 항목은 각각 질소 균형, 영양 지표, 회복 관련 임상 지표 등을 포함하며, 이에 대한 구체적 측정 항목과 평가 시점은 “유효성 평가 변수” 항목에서 별도로 상세 기술한다.

# 4. 임상시험을 통하여 예측되는 결과

1) 예측되는 주요 결과 (Expected Clinical Findings)

- 수술 후 5일째의 질소 균형(nitrogen balance, g/day)**이 시험군(위너프에이플러스군)에서 대조군(기존 TPN)보다 유의하게 높을 것으로 예측되며, 다양한 영양학적 지표의 개선(염증성 영양 지표 및 CT 기반 근육량 측정 등)을 보일 가능성이 높다.

- 수술 후 고혈당 발생률이 낮아지고, 혈당 스파이크의 빈도 및 폭이 줄어들 가능성이 있다.

2) 학술적 기대 효과 (Academic and Clinical Significance)

- 위암 수술 후 TPN 처방에서 고단백 정맥영양제제의 적용 타당성에 대한 임상 근거 제공

- 위너프에이플러스주와 같은 고단백·저당 조성의 4세대 TPN 제제의 생리학적 우수성을 객관적인 수치로 입증

- 위암 환자의 수술 후 회복 및 면역력 유지에 있어 정맥영양요법의 역할 확대 가능성 제시

결론적으로, 본 연구를 통해 고단백 정맥영양요법의 임상적 유용성과 생리학적 효과가 검증될 경우, 수술 후 영양불량의 조기개입 전략으로서의 정맥영양제제 선택 기준을 확립하는 데 중요한 학술적 기여를 할 것으로 기대된다. 나아가 향후 다기관 연구 및 수술 후 영양 가이드라인 정립을 위한 기반 자료로 활용 가능하다.

# 5. 임상시험용 의약품의 정보

## 5.1 시험약

위너프에이플러스 주 (고아미노산 함유, 4세대 TPN, 1500ml/1000ml peripheral)

## 5.2 대조약

위너프 주 (기존 TPN, peripheral)

# 6. 임상시험 기간

IRB 승인일로부터 3년

# 7. 시험대상자

## 7.1 선정기준

1) 19세 이상 위암 수술 예정자

2) 연구 참여에 서면 동의한 자

## 7.2 제외기준

1) 조절되지 않는 중증 질환자(대상 부전성 당뇨, 6개월 이내 cerebrovascular event(급성심근경색, 뇌졸증, 폐색전증 등), 패혈증, 심부전 등)

2) 무작위배정 7일 이내 경정맥 영양 공급이 시행된 자.

3) 수술 전 검사 상, 심한 대사성 이상이 확인된 자.

TG > 400 mg/dL - ALT/AST > 3 x UNL, Bilirubin > 3 x UN, - Creatinine > 2 x UNL, HbA1c > 9.0%, K < 3.0 mEq/L, K > 6.0 mEg/L, Ca > 12.5 mg/dL, Na > 155 mmol/L, Mg > 3.0 mg/dL

4) 시험자가 판단할 때, 임상시험 대상자로 부적절한 자

*탈락 대상자: 수술 중/후 중증 합병증이 발생한 자(대량 출혈 등)

## 7.3 대상자 수 및 산출 근거

### 7.3.1 대상자 수

|  | 설명 |
| --- | --- |
| 군 별 대상자 수 | 55명 |
| 전체 군 개수 | 2군(시험군, 대조군) |
| 군 간 대상자 비율 | 1:1 |
| 전체 대상자 수 | 110명 |

### 7.3.2 산출 근거

본 연구의 1차 유효성 평가변수는 수술 후 5일째(Postoperative day 5, POD5)의 질소 균형(nitrogen balance, g/day)이다. 시험군(고아미노산 4세대 TPN 투여군)과 대조군(기존 3세대 TPN 투여군) 간의 POD5 시점 nitrogen balance의 평균 차이를 비교하는 것을 주요 분석으로 설정하였다.

본 연구와 동일한 중재 비교를 직접 다룬 선행 무작위배정연구는 없으나, 관련 선행 문헌 및 임상적 타당성을 바탕으로 두 군 간 평균 nitrogen balance의 차이를 3 g/day, 표준편차(Standard deviation, SD)를 5 g으로 가정하였다. 양측 유의수준 0.05, 검정력 80%를 적용할 때 필요한 최소 표본 수는 각 군 44명으로 산출되었다.

이후 수술 후 합병증 발생, 중도 탈락, 추적관찰 실패 등 현실적인 요인을 고려하여 20%의 탈락률(drop-out rate)을 반영하였고, 이에 따라 목표 등록수는 각 군 55명, 총 110명(시험군 55명, 대조군 55명)으로 설정하였다.

따라서 본 연구는 총 110명의 위암 수술 환자를 모집하여, 무작위배정(random allocation)을 통해 시험군과 대조군에 1:1 비율로 배정하고, POD5의 nitrogen balance를 주요 평가 변수로 분석할 예정이다.

|  | 설명 |
| --- | --- |
| Primary End point | 수술 후 5일째(Postoperative day 5, POD5)의 nitrogen balance (g/day) |
| 가설 | 시험군(고아미노산 4세대 TPN)의 POD5 nitrogen balance 변화량이 대조군(기존 TPN)보다 유의하게 크다 |
| Primary End point 분석방법 | Two-sample t-test (독립표본 t검정) |
| 참고문헌 | Bozzetti F. Perioperative total parenteral nutrition in malnourished gastrointestinal cancer patients: a randomized clinical trial. Clin Nutr. 2001;20(6):477–485. \| |
| 참고문헌 값 | 시험군 Reference : 0.0 ± 5.0 g/day (예상 평균 ± 표준편차) |
|  | 대조군 Reference : -3.0 ± 5.0 g/day |
| 검정력 | 80% |
| 유의수준 | 양측 5% |
| 중도탈락률 | 20% |
| 군 별 대상자 수 | 55명 |
| 전체 대상자 수(중도탈락 고려) | 110명  [{44 / (1 – 0.2)} × 2군] |

* 무작위 배정 절차

본 연구에서는 시험군(4세대 고단백 정맥영양수액제)과 대조군(기존 TPN 제제)의 효과를 공정하게 비교하기 위해, 블록 무작위 배정(Block Randomization)을 적용한다.

1. 무작위 배정 방법

예상되는 전절제(Total gastrectomy) 대상자의 비율이 낮아 위 절제 범위에 따라 층화할 경우 층별 표본이 불균형해지고 소규모 층에서 블록이 미완성되어 배정이 준결정적으로 될 수 있는 점을 고려하여, 층화 없이 단일 배정표를 이용한 블록 무작위 배정을 적용한다.

위 절제 범위(전절제 vs 부분절제), 기저 영양 상태 등 주요 예후 인자는 무작위 배정 시 층화하는 대신 통계 분석 단계에서 공변량 보정(공분산분석, ANCOVA)을 통해 통제한다.

※ Proximal gastrectomy 환자는 포함하지 않으며, 예정된 수술 범위 기준으로 배정하고 실제 수술 범위가 변경되더라도 ITT 원칙에 따라 분석한다.

2. 블록 무작위 배정 (Block Randomization)

가변 블록 크기(4, 6)를 무작위 순서로 사용하여 시험군과 대조군이 1:1로 균등하게 배정되도록 단일 배정표를 연구 시작 전에 사전 생성한다(고정 seed를 사용한 스크립트로 생성, 총 120건 순차 배정). 연구자는 환자가 연구 참여에 동의하고 적합성이 확인된 후, 배정표에서 순서대로 상단부터 하나씩 배정군을 지정한다.

# 8. 연구 방법

## 8.1 일정표

| **Evaluation** | | **스크리닝** | **치료기** | | | | |
| --- | --- | --- | --- | --- | --- | --- | --- |
| **Visit** | | **V1** | **V2** | **V3** | **V4** | **V5** | **V6** |
| **Week** | | **-1W ± 7d** | **0W** | **1M ± 7d** | **3M ± 2W** | **6M ± 2W** | **12M ± 4W** |
| **서면동의서 취득** | | X |  |  |  |  |  |
| **인구학적 정보^1)^** | | X |  |  |  |  |  |
| **병력/약물복용력** | | X |  |  |  |  |  |
| **정맥영양수액 투여력 확인^2)^** | | X | X | X | X | X | X |
| **선정/제외기준 확인^3)^** | | X | X |  |  |  |  |
| **실험실검사^4)^** | | X |  |  |  | X | X |
| **신체검사(신장, 체중, BMI), 활력징후)** | | X |  |  |  | X | X |
| **식이요법 교육** | |  | X |  |  |  |  |
| **무작위배정^5)^** | | X |  |  |  |  |  |
| **유효성**  **평가** | **Nitrogen balance** |  | X |  |  |  |  |
|  | **Plasma glucose** |  | X |  | X | X |  |
|  | **Sarcopenia index^6)^** | X |  |  | X | △ |  |
|  | **Nutritional index^7)^** | X | X | X | X | X |  |
|  | **기능적 회복 지표** |  | X | △ | △ | △ |  |
| **항암 치료 관련 지표 확인^8)^** | |  |  | X | △ | △ | △ |
| **이상반응확인** | |  | X | X |  |  |  |

1) 성별, 나이, 체중 등의 기본인구학적 정보를 조사한다.

2) 스크리닝(V1) 전 1주 이내에 정맥영양수액 투여 여부를 확인한다.

3) 스크리닝(V1) 검사 결과가 선정기준을 벗어난 경우, 시험자의 판단에 따라 해당항목에 대해 재스크리닝 할 수 있다.

4) 스크리닝(V1) 시점을 기준으로 2주 이내 실시한 검사 값이 있는 경우 대체 가능.

 혈액학 검사: CBC(Hb, WBC, Lymphocyte, Neutrophil count)

 혈액생화학적 검사: AST, ALT, serum Creatinine , rGT, uric acid, s-amylase, CK

5) 무작위배정은 블록 무작위 배정(Block Randomization)을 적용한다(층화하지 않음).

- 위 절제 범위(전절제/부분절제)는 배정 시 기록하여 분석 단계의 공변량 보정에 이용한다.

6) APCT를 통해 sarcopenia index를 평가한다.

7) NRS-2002, CRP-Albumin ratio(CAR), Modified Glasgow Prognostic Score(mGPS), Prognostic Nutritional Index(PNI), GLIM criteria 를 측정한다.

8) 수술 후 보조항암요법 시행 여부 및 시행 용량을 확인한다.

**본 일정표는 주요 방문 시점과 개략적 평가 구조를 제시한 것이며, 세부 평가 항목과 정확한 수행 시점은 첨부된 최종 평가 일정표(SPIRIT schedule)에 따른다.

## 8.2 치료 방법 및 투약 계획

본 임상시험은 위암으로 근치적 수술을 받은 환자를 대상으로, 수술 전후 기간 동안 고단백 정맥영양수액제의 적용 효과를 평가하기 위해 설계된 무작위배정 임상연구이다. 대상자는 시험군(4세대 고단백 수액)과 대조군(기존 3세대 TPN 투여군)으로 무작위배정되며, 연구 등록 후 지정된 다음의 일정에 따라 정맥영양요법을 적용받는다.

TPN은 수술 전 1일(POD -1)부터 수술 후 5일(POD 5)까지 총 7일 중 수술 당일(POD 0)을 제외한 6일간 투여하게 된다. 수술 당일은 마취 및 수술 관련 처치로 인해 TPN은 일시적으로 중단되며, 그 외 6일 동안 TPN을 투여한다. 모든 수액은 말초 정맥(peripheral vein)을 통해 주입되며, 별도의 중심정맥 접근은 필요하지 않다.

하루 수액 요구량 중 최소 1,500 mL 이상을 정맥영양수액으로 구성하며, 환자의 체중과 수술 후 상태에 따라 NST(영양지원팀) 및 연구자 판단 하에 세부 조정이 이루어진다.

모든 환자는 수액 투여 기간 동안 매일 혈당, 전해질, 간기능, 신장기능, 체중, 소변량 등을 모니터링하고, 임상 상태에 따라 수액 구성이나 투여량을 조정할 수 있다. 고혈당(혈당 ≥ 250 mg/dL), 간기능 이상, 정맥염 등 이상반응이 발생할 경우 연구자 판단 하에 중단 혹은 감량이 가능하다.

## 8.3 검사항목 및 목적

| 구분 | 검사항목 | 검사방법 | 목적 | 비고 |
| --- | --- | --- | --- | --- |
| 영약학적 지표 | Prealbumin/albumin/protein | 혈액검사 | 혈액 내 단백질 수치 확인 |  |
|  | Sarcopenia index | APCT | 근육량 변화 확인 |  |
|  | Urea nitrogen | 소변검사 | Nitrogen balance 확인 |  |
| 면역학적 지표 | CRP | 혈액검사 | 염증 수치 확인 |  |
|  | total lymphocyte count | 혈액검사 | 염증 수치 확인 |  |
| 임상학적 지표 | 입원 기간 | EMR 확인 | 회복 속도 확인 |  |
|  | 합병증 | EMR 확인 | 합병증 발생율 확인 |  |
|  | Plasma glucose | 혈액검사 | 수술 후 혈당 변화 확인 |  |
| 기타 | 경구 섭취량 | EMR 확인 | Protein intake 확인 |  |

# 9. 평가 항목

## 9.1 일차 유효성 평가변수

**수술 후 5일째(Postoperative Day 5)의 질소 균형(Nitrogen balance, g/day) 변화량:** Change in nitrogen balance on postoperative day 5, assessed by 24-hour urinary urea nitrogen and protein intake.

- **평가 시점:** POD5 아침 채뇨 및 24시간 질소배설 측정
- **평가 지표:** 질소 균형 = 단백질 섭취량(식이+수액) – 질소 배설량(요중 요소 질소)
- **비교 방법:** 군 간 비교 (시험군 vs 대조군)
- **평가 방법:** 평균 변화량 비교 (변화량 분석)

## 9.2 이차 유효성 평가변수

1)중장기 영양 지표의 변화(수술 후 1-12개월)

Sarcopenia index assessed by APCT

Nutritional indices: NRS-2002, CRP-Albumin ratio(CAR), Modified Glasgow Prognostic Score(mGPS), Prognostic Nutritional Index(PNI), GLIM criteria

Laboratory findings: albumin, prealbumin, protein 등

2)수술 후 환자의 혈당 변화 비교: 수술 후 혈당 180mg/dl 이상 발생률 비교(당뇨 유무에 따라 환자 층화), 수술 후 일정 시점에 주기적으로 혈당 변화를 측정하여 혈당 변화 정도를 비교. – 수술 후 고혈당 이벤트 발생 빈도 확인 및 인슐린 요구량 등 측정

3)수술 후 합병증 발생률 비교: DGE 및 감염 관련 합병증(SSI, pneumonia) 발생률 비교 - Clavien-Dindo grade ≥2

4)기능적 회복 지표: 경구 섭취량, 입원 기간, 보행 시점 및 통증 강도 등

## 9.3 안전성 평가변수

이상사례(Adverse Event), 약물이상반응(Adverse Drug Reaction), 중대한 이상사례(Serious AE)

TPN 투여와 관련된 전해질 이상, 간수치 상승, 고혈당 등을 모니터링 할 계획임: AST, ALT, BUN, Creatinine, Glucose, Electrolytes 등

* 이상사례 발생 시 24시간 이내 JW중외제약 PV팀 (safety@jw-pharma.co.kr) 보고 예정

## 9.4 기타 평가변수

NA(해당 사항 없음)

# 10. 통계 분석 방법

## 10.1 일차 유효성 평가

수술 후 5일째의 질소 균형은 두 군 간 평균 비교를 위해 two-sample t-test로 분석하며(분포 가정이 명백히 위반될 경우 Wilcoxon rank-sum test), 주요 예후 인자(위 절제 범위, 기저 영양 상태) 및 공급된 질소량을 보정한 공분산분석(ANCOVA)을 사전 지정 분석으로 함께 시행한다.

## 10.2 이차 유효성 평가

1) 연속형 변수(Albumin, Prealbumin 등):

- 군 간 비교: **two sample t-test** 또는 Wilcoxon rank-sum test
- 동일군 내 전후 비교: **paired t-test** 또는 Wilcoxon signed-rank test

2) 혈당 관련 지표:

- 고혈당 발생율 비교: **Chi-square test** 또는 **Fisher’s exact test**
- 혈당 평균 변화량: **t-test**

3) Sarcopenia Index 변화:

- 수술 전후 변화량 비교: paired t-test
- Sarcopenia 발생율 비교: **Chi-square test** 또는 **Fisher’s exact test**

4) 입원 기간, 합병증 발생 등: 군 간 비교 (t-test 또는 rank-sum test)

## 10.3 안전성 평가

이상사례는 군 별로 발현 대상자 수, 발현율, 총 건수를 제시하고,

- 군 간 **발현율 비교는 Chi-square test 또는 Fisher’s exact test**로 수행
- 이상사례 발생 시점, 중대성, 중단 여부 등은 기술통계로 요약
  10.4 기타 평가

NA(해당 사항 없음)

# 11. 참고문헌

1.	Bozzetti F, Braga M, Gianotti L, Gavazzi C, Mariani L. Postoperative enteral versus parenteral nutrition in malnourished patients with gastrointestinal cancer: a randomised multicentre trial. Lancet. 2001;358(9292):1487-92.

2.	Pressoir M, Desne S, Berchery D, Rossignol G, Poiree B, Meslier M, et al. Prevalence, risk factors and clinical implications of malnutrition in French Comprehensive Cancer Centres. Br J Cancer. 2010;102(6):966-71.

3.	Hebuterne X, Lemarie E, Michallet M, de Montreuil CB, Schneider SM, Goldwasser F. Prevalence of malnutrition and current use of nutrition support in patients with cancer. JPEN J Parenter Enteral Nutr. 2014;38(2):196-204.

4.	Muscaritoli M, Lucia S, Farcomeni A, Lorusso V, Saracino V, Barone C, et al. Prevalence of malnutrition in patients at first medical oncology visit: the PreMiO study. Oncotarget. 2017;8(45):79884-96.

5.	Veterans Affairs Total Parenteral Nutrition Cooperative Study G. Perioperative total parenteral nutrition in surgical patients. N Engl J Med. 1991;325(8):525-32.

6.	Weimann A, Braga M, Carli F, Higashiguchi T, Hubner M, Klek S, et al. ESPEN practical guideline: Clinical nutrition in surgery. Clin Nutr. 2021;40(7):4745-61.

7.	Yun JH, Song GJ, Son MW, Lee MS. Global Leadership Initiative on Malnutrition Criteria and Immunonutritional Status Predict Chemoadherence and Survival in Stage II/III Gastric Cancer Treated with XELOX Chemotherapy. Nutrients. 2024;16(20).

8.	Arends J, Baracos V, Bertz H, Bozzetti F, Calder PC, Deutz NEP, et al. ESPEN expert group recommendations for action against cancer-related malnutrition. Clin Nutr. 2017;36(5):1187-96.

9.	Arends J, Strasser F, Gonella S, Solheim TS, Madeddu C, Ravasco P, et al. Cancer cachexia in adult patients: ESMO Clinical Practice Guidelines(☆). ESMO Open. 2021;6(3):100092.

10.	Bauer J, Biolo G, Cederholm T, Cesari M, Cruz-Jentoft AJ, Morley JE, et al. Evidence-based recommendations for optimal dietary protein intake in older people: a position paper from the PROT-AGE Study Group. J Am Med Dir Assoc. 2013;14(8):542-59.

11.	Hoffer LJ, Bistrian BR. Appropriate protein provision in critical illness: a systematic and narrative review. Am J Clin Nutr. 2012;96(3):591-600.

12.	Cotogni P, Shaw C, Jimenez-Fonseca P, Partridge D, Pritchett D, Webb N, et al. High-protein home parenteral nutrition in malnourished oncology patients: a systematic literature review. Support Care Cancer. 2023;32(1):52.

13.	Yamashita K, Yamasaki M, Miyazaki Y, Matsuura N, Tanaka K, Makino T, et al. Protein-enhanced feeds after esophagectomy for esophageal cancer attenuate postoperative catabolism: a prospective observational study. Surg Today. 2022;52(4):624-32.

14.	Okamoto Y, Sakaguchi T, Ikematsu Y, Kanai T, Hirayama K, Tamura H, et al. Early enteral nutrition with arginine compensates for negative nitrogen balance in patients undergoing curative total gastrectomy. J Med Invest. 2023;70(3.4):325-33.
