# Supplementary material for: The PROGAIN trial: A randomized controlled trial of high-protein peripheral parenteral nutrition on nitrogen balance and recovery after gastric cancer surgery — study protocol
Source: PLoS One. 2026 Aug 7;21(8):e0355677. doi: 10.1371/journal.pone.0355677 (PMC13450730; doi:10.1371/journal.pone.0355677)
Supplement: Supplementary file 3 [file pone.0355677.s011.docx]

**Response to Reviewers**

Manuscript: PONE-D-26-26899

Title: The PROGAIN trial: a randomized controlled trial of high-protein peripheral parenteral nutrition on nitrogen balance and recovery after gastric cancer surgery — study protocol

We thank the Academic Editor and the four reviewers for their careful and constructive evaluation of our protocol. We have revised the manuscript accordingly and respond to each point below. Reviewer comments are shown in italics, followed by our response. Each reviewer’s responses are self-contained; where two reviewers raised a similar point, we provide a full answer under each. Changes are marked in the tracked-changes version of the manuscript.

**Journal Requirements (Academic Editor)**

**1.** *Please ensure the manuscript meets PLOS ONE style requirements, including file naming.*

**Response.** The manuscript, title page, and file names have been reformatted to conform to the PLOS ONE templates and file-naming conventions.

**2.** *Registration appears to have occurred after recruitment began. Please state in the Methods (i) the reasons for the delay, and (ii) the confirmation that all related trials are registered.*

**Response.** The Trial status section now states the reason for the delayed registration (administrative processing and access to the institutional registration system following IRB approval) and includes the required statement: “The authors confirm that all ongoing and related trials for this drug/intervention are registered.” The Trial status section additionally documents that the IRB-approved protocol and the outcomes and statistical analysis plan were finalized before the first participant was enrolled and that the registry record corresponds to that protocol.

**3.** *You received funding from a commercial source (JW Pharmaceutical). Please provide an amended Competing Interests statement and confirm adherence to PLOS data/materials-sharing policies.*

**Response.** The Competing Interests statement (provided in the cover letter for entry into the submission system) now explicitly names the commercial funder and reads: “This study was funded in part by JW Pharmaceutical, a commercial source, which manufactures both the investigational and comparator products. The coordinator salary and the article-processing charge were supported by JW Pharmaceutical. The funder had no role in study design, data collection and analysis, interpretation, or manuscript preparation. This does not alter our adherence to PLOS ONE policies on sharing data and materials.”

**4.** *Funding information should not appear in the manuscript; it will be published only from the Funding Statement in the submission system.*

**Response.** All funding-related text has been removed from the manuscript body; the funding information is provided solely in the Funding Statement of the online submission form.

**5.** *Cite reviewer-recommended works only where relevant.*

**Response.** We evaluated the suggested topics and added citations only where they strengthen specific statements (see Reviewer 2, Comments 1 and 7).

**Reviewer #1**

*The design and analysis strategy are sufficiently outlined; the study is well planned and presented, and Table 4 is an excellent presentation of the analysis strategy. (No revisions requested.)*

**Response.** We are grateful to the reviewer for the positive assessment and encouraging comments.

**Reviewer #2**

**2-1.** *Routine PN, especially on the day before surgery, is not standard in the West, and Western partial-gastrectomy stays are often shorter than 5 postoperative days. Please comment on how often standard perioperative PN (including the day before surgery) is used in Korea and other Asian settings.*

**Response.** We agree this context is important and provide the following clarification in response; because the reviewer raises this as a question and the point does not require a change to the protocol, we address it here rather than in the manuscript. Routine perioperative PN, including administration on the day before surgery, is common practice in Korea and other East Asian countries and differs from enhanced-recovery–dominant Western practice. Postoperative length of stay after gastrectomy in East Asia is substantially longer than the sub–five-day stays sometimes reported in Western partial gastrectomy: recent two-centre and multicentre analyses from Korea, China, and Japan report mean postoperative stays of approximately 8.6–9.0 days. In a Chinese multicentre ERAS cohort of 1,434 patients, the mean time to first flatus was 4.4 days and the mean time to initiation of a liquid diet was 6.1 days, indicating that adequate oral intake is typically not achieved until after POD 5. In addition, admission on the day before surgery is common in this setting for indocyanine green injection for sentinel node mapping and surgical marking, which is why PN begins on preoperative day −1. Within this context, a six-day perioperative PN course and a POD 5 nitrogen-balance endpoint fall well within the usual inpatient trajectory, while the mechanistic findings are expected to generalize beyond it. Supporting citations have been added.

**2-2.** *There is no mention of nitrogen balance as an appropriate proxy for nutritional status/protein intake. What data support this? Have clinically meaningful differences been established? What is the significance of a 3 g/day difference?*

**Response.** Nitrogen balance is an objective, quantifiable, blinded-assay marker of net protein metabolism — the difference between nitrogen intake and nitrogen loss — and has been used as a primary or principal endpoint in perioperative and nutritional randomized trials. It is not simply a proxy that rises automatically with intake: in the catabolic state after major surgery, additional amino acids are frequently oxidized and excreted rather than retained, and several randomized trials of enriched amino-acid regimens have shown no improvement in nitrogen balance despite greater or altered amino-acid delivery. Demonstrating an improvement therefore reflects genuine net retention rather than arithmetic. Regarding clinical meaning, a between-arm difference of 3 g/day in nitrogen balance corresponds to approximately 19 g/day of protein-equivalent and represents a clinically meaningful shift during the early postoperative catabolic period, comparable in magnitude to differences reported in prior perioperative protein-supplementation studies. To make the interpretation explicit, we have added prespecified mechanistic analyses that decompose the endpoint into total nitrogen intake, urinary urea nitrogen excretion, and net nitrogen retention, together with a nitrogen-utilization analysis. Supporting citations have been added.

**2-3.** *Wouldn’t giving more protein necessarily change nitrogen balance? Why not select more clinically relevant endpoints?*

**Response.** We selected nitrogen balance as the primary endpoint because it is an objective, quantifiable, blinded-assay outcome that is comparatively resistant to the ascertainment bias to which subjective or clinically graded outcomes are prone in an open-label, single-centre trial, and because it directly reflects the mechanism of the intervention (protein and nitrogen economy) on a clinically relevant short timescale; clinically oriented recovery outcomes require much larger samples to assess reliably and are captured comprehensively as prespecified secondary outcomes. We agree that a difference in nitrogen balance is meaningful only if it reflects genuine retention rather than intake alone. To address this, we have added prespecified mechanistic analyses decomposing total nitrogen intake, urinary urea nitrogen, and net retention, together with a utilization-efficiency analysis; notably, the between-arm difference in delivered nitrogen on POD 5 is only approximately 1.0–1.2 g/day, so an observed nitrogen-balance difference of the assumed 3 g/day could not be explained by intake alone and would indicate a genuine metabolic effect. The interpretation and its limitations are discussed in the revised manuscript.

**2-4.** *Are all gastrectomy types (wedge, distal/subtotal, proximal, total), reconstructions (Roux-en-Y, Billroth I/II, double tract), and approaches (open, laparoscopic, robotic) included? Please specify, and note that heterogeneity may limit interpretation and subgroup feasibility.*

**Response.** We have clarified eligibility. Eligibility is based on the planned extent of resection: planned total and distal (subtotal) gastrectomy are eligible, whereas planned proximal gastrectomy is excluded (now stated explicitly in Table 1). All standard reconstructions (Billroth I, Billroth II, Roux-en-Y) and surgical approaches (open, laparoscopic, robotic) are permitted. If the actual procedure differs from the plan (including an unplanned proximal gastrectomy), the participant is retained and analyzed in the allocated arm under the intention-to-treat principle, with the actual procedure recorded and examined in per-protocol/sensitivity analyses. We have added a statement acknowledging that heterogeneity of resection type may complicate interpretation and that subgroup analyses are exploratory and may be underpowered.

**2-5.** *How is PN handled when patients develop anastomotic leak and/or bacteremia or fungemia?*

**Response.** For severe postoperative complications, including anastomotic leak, bacteremia, or fungemia, the allocated PN is discontinued at the discretion of the treating clinician; such participants are retained in the intention-to-treat population, excluded from the per-protocol population, and flagged for sensitivity analysis. This is now stated in the Interventions (Monitoring and dose modification) and Safety sections.

**2-6.** *Will patients with cirrhosis or chronic kidney disease be included, given the risks of a high-protein regimen?*

**Response.** Patients with significant hepatic or renal impairment are excluded by the existing screening thresholds (AST or ALT > 3× ULN, total bilirubin > 3× ULN, serum creatinine > 2× ULN), which exclude decompensated hepatic disease and clinically significant chronic kidney disease. Hepatic and renal function, blood urea nitrogen, glucose, and electrolytes are monitored daily during the intervention, with predefined safety triggers for dose reduction or discontinuation. We have made this explicit in the Eligibility and Safety sections.

**2-7.** *Introduction (lines 41–42): please provide citations for the stated outcome associations.*

**Response.** Citations have been added for the associations between inadequate perioperative protein supply and delayed wound healing, infectious complications, prolonged hospital stay (perioperative nutrition literature), and impaired adjuvant chemotherapy tolerance (our prior institutional data).

**Reviewer #3 — Major comments**

**1.** *The primary endpoint is partly determined by the intervention itself; a fraction of the assumed effect could be explained by intake difference alone. State the question of interest (retention vs utilization), discuss this limitation, and prespecify separate analyses of (a) total nitrogen intake, (b) urinary urea nitrogen, and (c) net nitrogen retention, ideally with delivered nitrogen as a covariate or a utilization-efficiency analysis.*

**Response.** We thank the reviewer for this important point, which we have addressed in three ways. First, we have clarified the interpretation: nitrogen balance is presented as a clinically oriented marker of net nitrogen retention that is, by design, partly dependent on nitrogen intake, rather than as a fully independent biological endpoint. Second, we emphasize that an improvement is not a foregone conclusion: in catabolic surgical patients, additional amino acids are frequently oxidized and excreted rather than retained, and several randomized trials of enriched amino-acid regimens have found no improvement in nitrogen balance; the trial therefore tests whether this formulation produces genuine net retention. Third, we have added prespecified mechanistic secondary analyses that decompose the endpoint into (a) total nitrogen intake, (b) urinary urea nitrogen excretion, and (c) net nitrogen retention, together with a nitrogen-utilization analysis expressed as net retention relative to delivered nitrogen and, equivalently, as an analysis of the primary endpoint adjusting for delivered nitrogen as a covariate (integrated into the ANCOVA described in Major 5). Because the between-arm difference in delivered nitrogen on POD 5 is only about 1.0–1.2 g/day (see Major 6), an observed nitrogen-balance difference of the assumed magnitude could not be explained by intake alone and would indicate a genuine difference in retention/utilization. A corresponding limitation has been added.

**2.** *The nitrogen-balance formula should use exact parenteral nitrogen delivery, not a 6.25 conversion; Table 2 provides product-specific nitrogen content.*

**Response.** We agree and have revised the formula. Nitrogen balance is now computed as [oral protein (g) ÷ 6.25 + parenteral nitrogen delivered (g)] − (urinary urea nitrogen (g) + 4), where parenteral nitrogen is derived directly from the product-specific nitrogen content in Table 2 (5.21 mg/mL for the control and 6.17 mg/mL for the investigational product) multiplied by the administered volume, rather than by dividing amino-acid grams by 6.25. The 6.25 factor is retained only for dietary (oral) protein. This exact accounting is applied consistently and underlies the mechanistic decomposition in Major 1.

**3.** *Feasibility and quality control of the 24-hour urine collection require detail: collection window, missed voids, completeness validation, discharge before POD 5, and drain/NG/GI losses; note the +4 g constant limitation.*

**Response.** We have added operational detail. The 24-hour urinary urea nitrogen collection is performed over POD 5 (00:00–24:00) by self-voided collection, as urinary catheters are removed early under the enhanced-recovery pathway; participants receive standardized instruction (discarding the first morning void and including the final void at hour 24), and collection start and end times are documented. Total 24-hour urine volume, which is recorded for every participant, is used as a plausibility check, and any missed or incomplete void is flagged; collections judged incomplete are handled per the missing-data plan (Major 7). Because all participants remain hospitalized through POD 5 in this population, discharge before the primary assessment is not anticipated. Participants with clinically significant non-urinary gastrointestinal losses (high-output drainage, prolonged nasogastric aspiration, substantial vomiting, or anastomotic leak) are flagged and examined in a prespecified sensitivity analysis. We have added a limitation stating that the fixed +4 g constant does not capture surgical gastrointestinal losses and that completeness was not verified by creatinine excretion; because both apply similarly across the randomized arms, they are expected to affect the absolute nitrogen balance more than the between-arm comparison, and residual collection error is expected to be non-differential.

**4.** *The intervention is a composite (amino acids, dextrose, NPC/N, electrolytes, osmolarity, and volume all differ). Present the expected daily delivered amounts for both arms at the scheduled coverage on each POD, and discuss the composite nature.*

**Response.** We agree. A new supporting table (S_ Table) presents the expected daily delivered amounts of amino acids, nitrogen, glucose, and lipid (with total volume) for both arms at the scheduled coverage on each intervention day (POD −1 to POD 5); electrolytes scale proportionally with volume, as noted in the table footnote. The Discussion now states explicitly that the investigational formulation simultaneously delivers higher protein, lower carbohydrate, and a lower NPC/N ratio, and that the glycemic secondary outcomes therefore partly reflect the difference in carbohydrate load rather than a high-protein effect alone; this compositional rationale was intentional and is now made explicit.

**5.** *Stratified randomization is not used, yet a subgroup analysis by extent of resection is planned. Justify non-stratification or adopt it; at minimum prespecify an adjusted (ANCOVA) primary analysis for key prognostic covariates.*

**Response.** We appreciate this comment and have both clarified and strengthened the analysis. Stratified randomization was not used because the anticipated low proportion of total gastrectomy would produce imbalanced strata and incomplete blocks in the small stratum, making non-stratified block randomization with covariate adjustment preferable; key prognostic factors (extent of resection and baseline nutritional status) are instead controlled analytically. We have prespecified an ANCOVA-adjusted analysis of the primary endpoint that includes extent of resection, baseline nutritional status, and delivered nitrogen as covariates (also serving the utilization analysis in Major 1). We note transparently that the IRB-approved protocol had described stratified randomization; because the trial has consistently used non-stratified block randomization since its start, we have submitted an IRB amendment (version 1.2) so that the protocol, the registry record, and the manuscript are consistent; this amendment is currently under IRB review, with a decision expected on 15 July 2026, and affects neither eligibility, the intervention, the endpoints, nor the target sample size. We will provide the approval notice once issued.

**6.** *The assumed effect size (3 g/day, SD 5) should be better justified given that scheduled POD 5 PN coverage is only 50%; comment on sensitivity to the assumed SD.*

**Response.** We have added a quantitative rationale and a sensitivity analysis. Using the product-specific nitrogen content and the scheduled coverage, the between-arm difference in delivered nitrogen is approximately 1.0–1.2 g/day on POD 5 and larger earlier in the intervention (up to ~2 g/day on POD 1), giving a cumulative delivered-nitrogen difference of roughly 8 g over the six-day course; the nitrogen balance on POD 5 reflects this cumulative metabolic exposure rather than a single day’s intake. Because the assumed 3 g/day balance difference exceeds the single-day delivered-nitrogen difference, detecting it would require a genuine difference in retention rather than arithmetic intake alone (linking to Major 1). We have added a power-sensitivity table showing power under a range of effect sizes (Δ = 2.0, 2.5, 3.0 g/day) and standard deviations (SD = 4, 5, 6 g/day), and we acknowledge in the limitations that the trial may be underpowered if the true effect is smaller than assumed, in which case the mechanistic analyses remain informative.

**7.** *Missing-data handling is too vague. Prespecify the primary estimand and the primary missing-data strategy for incomplete POD 5 collection, early discharge, complications, death, PN discontinuation, or withheld PN, and add MNAR sensitivity analyses.*

**Response.** We have replaced the vague statement with a prespecified estimand and strategy following ICH E9(R1). The primary estimand targets the between-arm difference in mean POD 5 nitrogen balance in the intention-to-treat population using a treatment-policy strategy for intercurrent events: temporary or permanent PN dose reduction/discontinuation is handled by using the observed POD 5 value (which reflects the actual delivered nitrogen), so these are not treated as missing. Genuine missing data arise only from death before POD 5 or failed/incomplete collection; for these, the primary approach is multiple imputation under a missing-at-random assumption (imputation model including arm, baseline covariates, and auxiliary variables such as POD 3 nitrogen balance), with complete-case analysis as a sensitivity analysis. Because missingness related to postoperative complications may not be missing-at-random, we have added an MNAR sensitivity analysis using a delta-adjusted (tipping-point) approach.

**8.** *Multiplicity control is not prespecified. Distinguish key secondary from exploratory outcomes and commit to a specific strategy.*

**Response.** We have specified a clear structure. The single primary endpoint (POD 5 nitrogen balance) is the sole confirmatory analysis (two-sided α = 0.05). A small set of key secondary endpoints — POD 3 nitrogen balance and early change in prealbumin, both objective, blinded, and responsive to short-term protein delivery — is controlled for family-wise error using the Holm procedure (not gated to the primary, so a key secondary can be declared significant independently). All other outcomes, including postoperative complications, glycemic outcomes, skeletal muscle index, functional recovery, patient-reported outcomes, and the mechanistic nitrogen analyses, are exploratory and are reported with point estimates and 95% confidence intervals without multiplicity adjustment and interpreted as hypothesis-generating; postoperative complications are additionally examined with multivariable adjustment for surgical and comorbidity factors. This estimation-based approach for exploratory outcomes is stated explicitly.

**9a.** *Open-label design: oral protein intake, which enters the numerator, is recorded by unblinded staff/patients. Clarify who adjudicates complications and whether they are blinded, and whether standardized discharge and diet-advancement criteria are applied; state the intake-recording limitation.*

**Response.** We have clarified these points. Oral intake is recorded as a patient-reported outcome: after standardized in-hospital education, participants complete a meal-by-meal intake form, which is verified by the nutrition support team; we now state explicitly that this self-reported intake is a potential source of ascertainment bias in an open-label trial, which was one reason for selecting an objective, blinded assay (urinary urea nitrogen) for the primary endpoint. Postoperative complications are graded using the Clavien–Dindo classification by an independent assessor who is blinded to group allocation, and standardized institutional discharge and diet-advancement (enhanced-recovery) criteria are applied to reduce subjectivity. The residual potential for bias in self-reported intake and in clinically graded outcomes is acknowledged in the limitations.

**9b.** *Registration: state whether any participant completed the POD 5 primary endpoint before registration, whether the registry record matches the IRB-approved protocol, and whether outcomes/eligibility/schedule/analysis changed between IRB approval, first enrollment, and registration.*

**Response.** We have addressed this fully and transparently. The trial received IRB approval on 5 December 2025, the first participant was enrolled on 13 February 2026, and the trial was registered at ClinicalTrials.gov on 14 March 2026, approximately 29 days after first enrollment; we acknowledge that this constitutes retrospective registration. The IRB-approved protocol and the sponsor agreement were finalized before the first participant was enrolled, and the registry record corresponds fully to the IRB-approved protocol, with no change to eligibility, the intervention, the primary or secondary outcomes, or the statistical analysis plan. By the registration date, four participants had passed POD 5 by calendar date; a confirmed POD 5 primary-endpoint result was available for three of them (the fourth, operated on 6 March, did not yet have a confirmed result, as 14 March was a weekend). Because the outcomes and the analysis plan had been fixed in the IRB-approved protocol before enrollment, the availability of these three early results did not permit any post hoc selection or modification of outcomes or analyses. As noted in Major 5, the protocol description of randomization has been submitted as an IRB amendment (v1.2) revising stratified to non-stratified block randomization with prespecified covariate adjustment; this amendment is currently under IRB review, with a decision expected on 15 July 2026, and affects neither eligibility, the intervention, the endpoints, nor the target sample size. All future related trials will be registered prospectively.

**Reviewer #3 — Minor comments**

**1.** *Table 4 cites “Loder et al., 1989,” which is not in the reference list; the text uses Blackburn 1977 and Dickerson 2005.*

**Response.** The erroneous citation has been removed; the nitrogen-balance formula is referenced consistently to Blackburn 1977 and Dickerson 2005 throughout.

**2.** *“354 inpatients” versus a Table 3 footnote of 92+160+102 = 354 measurements.*

**Response.** This was an inconsistency in wording. The audit comprised 354 stage-level meal-intake measurements (92, 160, and 102 for the liquid, early soft, and soft diet stages); the text and the S1 Table title now consistently refer to “354 measurements” rather than to patients.

**3.** *In Table 3, the POD 3 early soft diet (~315 kcal/day) is lower than the POD 2 liquid diet (~350 kcal/day), which is counterintuitive.*

**Response.** This pattern is real rather than an error. The liquid-diet stage is supplemented with carbohydrate drinks, so its total energy slightly exceeds that of the early soft stage, which relies on lower-energy soft foods during the transition; the same non-monotonic pattern is present in the standardized provided diet (approximately 510 vs 480 kcal/day). A clarifying sentence has been added.

**4.** *Diet-stage terminology is inconsistent (“soft diet” vs “congee diet”; awkward phrasing).*

**Response.** Diet-stage terminology has been standardized throughout to “liquid,” “early soft,” and “soft” diet.

**5.** *POD −1 is potentially confusing.*

**Response.** “Preoperative day −1” is now used consistently on first use, with “POD −1” thereafter.

**6.** *The introduction emphasizes that conventional PN delivers ~0.9 g/kg/day (below the ESPEN minimum), but nitrogen balance is computed from total protein.*

**Response.** We now distinguish PN-only protein delivery (~0.9 g/kg/day for the control formulation) from total protein supply (oral plus parenteral), to avoid implying undertreatment and to preserve equipoise.

**7.** *Clarify whether oral nutritional supplements are permitted during POD 1–5, and how they are standardized, recorded, and incorporated into the nitrogen-balance calculation.*

**Response.** Oral nutritional supplements beyond the protocol-specified standardized perioperative diet are not permitted during the intervention period. All oral intake, including the standardized supplement components of the diet, is recorded on the meal-by-meal intake form and included in the oral-protein term of the nitrogen-balance calculation. This is now stated in the Interventions section.

**8.** *The Fig 2 legend states skeletal muscle index is measured on POD 5, whereas the Outcomes section and Table 4 specify baseline and follow-up CT.*

**Response.** The Fig 2 legend has been corrected to indicate that the skeletal muscle index is assessed from baseline and follow-up computed tomography, consistent with the Outcomes section and Table 4.

**9.** *Data availability states “no datasets generated or analyzed,” which conflicts with the existing institutional audit data (S1 Table, n = 354).*

**Response.** The data-availability statement has been revised to reflect that the institutional audit dataset underlying S1 Table exists and to describe access conditions; no participant outcome data have yet been generated for this ongoing protocol.

**10.** *Specify the rationale and applicable range for the ideal-body-weight formula (height² × 23, at BMI ≥ 25).*

**Response.** The reference body-mass index of 23 kg/m² reflects WHO recommendations for Asian populations; a citation and a brief rationale, with the applicable range (applied when BMI ≥ 25 kg/m²), have been added.

**11.** *Given the higher osmolarity and the manufacturer’s involvement, describe peripheral line management, phlebitis grading, thresholds, insulin management, and stopping rules, and justify the absence of a DSMB.*

**Response.** The Safety section now states that infusion-site reactions are graded using the Visual Infusion Phlebitis score and that peripheral catheters are re-sited per standard nursing practice; predefined safety triggers (blood glucose ≥ 250 mg/dL with protocol-directed insulin management, clinically significant hepatic enzyme elevation, and infusion-site reactions) and stopping/discontinuation rules are specified. No formal data and safety monitoring board was convened given the single-centre design, the short six-day intervention, and the use of two regulatory-approved PN products with established safety profiles; safety is overseen by the principal investigator with periodic IRB review.

**12.** *The financial disclosure should include grant numbers, initials of funded authors, and funder URLs.*

**Response.** Per the Academic Editor’s Requirement 4, all funding information has been removed from the manuscript body and is provided in full — including funder details and the supported activities — in the Funding Statement of the submission system.

**13.** *In the compiled reviewer PDF, the figures, SPIRIT checklist, and IRB protocol appeared as placeholders rather than readable content.*

**Response.** We have re-uploaded all figures and supporting files (figures, SPIRIT checklist, and IRB-approved protocol) in the required formats so that they render legibly in the compiled PDF.

**Reviewer #4**

**1.** *Please add clarification on registration transparency: whether the retrospective registration was reported to the registry platform, whether the full protocol received IRB approval before the first enrollment, and whether the primary endpoints and SAP were finalized before registration without modification.*

**Response.** We have clarified these points. The full protocol received IRB approval on 5 December 2025, before the first participant was enrolled on 13 February 2026. The primary and secondary endpoints and the statistical analysis plan were finalized in the IRB-approved protocol before enrollment and before registration, without modification; the registry record corresponds to that protocol. We disclose transparently that public registration occurred on 14 March 2026, after first enrollment (approximately 29 days later), that this constitutes retrospective registration, and that the registry record has been completed and updated; all future related trials will be registered prospectively.

**2.** *Provide the rationale for the 20% dropout assumption in the sample-size calculation.*

**Response.** We have added a rationale. The 20% allowance reflects the upper end of attrition typically assumed in comparable perioperative and surgical-nutrition randomized trials and is appropriate given the 12-month follow-up in a gastric-cancer surgical population (in which adjuvant therapy, recurrence, and loss to follow-up contribute to attrition). A supporting citation has been added.

**3.** *Please describe the unblinding procedure.*

**Response.** We have added that the coded group labels (Arm A / Arm B) will be unmasked by an independent individual only after database lock and finalization of the statistical analysis plan, completing the blinding workflow.

We thank the editor and reviewers again for their constructive comments, which have substantially improved the manuscript.
