# Supplementary material for: The PROGAIN trial: A randomized controlled trial of high-protein peripheral parenteral nutrition on nitrogen balance and recovery after gastric cancer surgery — study protocol
Source: PLoS One. 2026 Aug 7;21(8):e0355677. doi: 10.1371/journal.pone.0355677 (PMC13450730; doi:10.1371/journal.pone.0355677)
Supplement: Supplementary file 4 [file pone.0355677.s012.docx]

**Response to Reviewers (Revision 2)**

Manuscript: PONE-D-26-26899R1

Title: The PROGAIN trial: a randomized controlled trial of high-protein peripheral parenteral nutrition on nitrogen balance and recovery after gastric cancer surgery — study protocol

We thank the Academic Editor and the reviewers for the positive assessment. Reviewers 1, 2, and 4 indicated that their comments were fully addressed, and Reviewer 4 recommended acceptance. Reviewer 3 raised six remaining points for clarification, which we address below. Reviewer comments are in italics; changes are marked in the tracked-changes manuscript.

**Academic Editor**

*Please address the reviewer’s minor revision comments before final submission.*

**Response.** We have addressed all six of Reviewer 3’s points, as detailed below. No changes to the financial disclosure are made; the cover letter and submission-system statements are unchanged.

**Reviewer #1, #2, and #4**

**Response.** We are grateful for the confirmation that our previous revisions resolved the reviewers’ comments and for Reviewer 4’s recommendation of acceptance. No further changes were requested by these reviewers.

**Reviewer #3**

**1.** *Protocol v1.2 (stratified → non-stratified randomization) remains under IRB review. Provide the final IRB approval and the approved v1.2 with English translation, and clarify how many participants were randomized before this amendment and how the discrepancy was formally handled.*

**Response.** Protocol amendment version 1.2 was approved by the Institutional Review Board of Soonchunhyang University Cheonan Hospital on 16 July 2026 (SCHCA IRB 2025-11-024-003). We now provide the IRB approval notice (Korean original with an English translation) as an “Other” file, and the approved protocol version 1.2 with its English translation as S1 Protocol. We have also clarified the conduct: the trial has used non-stratified block randomization consistently from the first participant, and all participants randomized to date were allocated by this single, unchanged method. The protocol document had described stratified randomization; the amendment therefore corrects the protocol document to match the actual, unchanged conduct rather than changing the randomization method, and it affects neither eligibility, the intervention, the endpoints, nor the target sample size. The Trial status section has been updated accordingly.

**2.** *The Table 4 footnote is inconsistent with the revised Statistical Analysis section (still refers to hierarchical/FDR and to MI only when missingness exceeds 5%). Revise it to reflect the Holm procedure, the MAR-based MI strategy, and the MNAR tipping-point analysis, and identify the two key secondary outcomes and the exact definition and timepoint of “early change in prealbumin” in Table 4.*

**Response.** The Table 4 footnote has been rewritten to match the Statistical Analysis section. It now states that the single primary endpoint is the sole confirmatory analysis; that the two key secondary endpoints — POD 3 nitrogen balance and the early change in serum prealbumin, defined as the change from the preoperative (POD −1) baseline to POD 5 — are controlled for family-wise error by the Holm procedure independently of the primary result; and that the primary-endpoint missing data are handled by MAR-based multiple imputation with complete-case and MNAR delta-adjusted (tipping-point) sensitivity analyses. The two key secondary outcomes and the prealbumin definition/timepoint are now identified in the footnote.

**3.** *Clarify the dates and version numbers of the amended protocol and SAP incorporating the ANCOVA, mechanistic analyses, multiplicity procedure, and missing-data analyses; these should not be described simply as “prespecified” unless documented in a dated protocol or SAP, finalized before any aggregate or between-group analysis.*

**Response.** We have prepared a dated Statistical Analysis Plan (SAP version 1.0, 21 July 2026) documenting the covariate-adjusted ANCOVA, the exploratory mechanistic nitrogen analyses, the multiplicity procedure, and the missing-data strategy; it is provided as a supporting file (S2 File) and is cited in the Trial status section and the Table 4 footnote. The SAP states, and we confirm, that it was finalized before any aggregate or between-group analysis of trial data (the trial is recruiting and no such analysis has been performed). The covariate-adjusted ANCOVA is additionally documented in the IRB-approved protocol version 1.2 (approved 16 July 2026). The manuscript now cites the dated SAP and protocol version so that these analyses are supported as genuinely prespecified.

**4.** *The Data Sharing Statement says no participant outcome datasets have been generated, whereas the Trial Status section states that confirmed POD 5 results were available for three participants. Revise to clarify that outcome data are being collected but no aggregate or treatment-group analysis has been performed.*

**Response.** The Data Sharing Statement has been revised to read that participant outcome data are being collected in this ongoing trial but that no aggregate or treatment-group analysis has been performed, resolving the inconsistency with the Trial status section.

**5.** *Correct remaining inconsistencies: “congee diet” in Table 3, the extraneous citation “[9]” at line 196, inconsistent citation formatting, and the 20% sample-size inflation rationale (should relate to missingness of the POD 5 primary outcome, not 12-month attrition).*

**Response.** All items have been corrected. “Congee diet” in Table 3 is now “soft diet,” consistent with the standardized terminology. The extraneous “[9]” has been removed. Citation formatting has been unified to the numeric style throughout, including the nitrogen-balance formula citation in Table 4 (now numeric). The rationale for the 20% inflation has been rewritten to relate specifically to missingness of the POD 5 primary outcome (incomplete 24-hour collection, early events, PN discontinuation, or death before POD 5) rather than to attrition during the 12-month follow-up.

**6.** *Because delivered nitrogen is a post-randomization variable largely determined by allocation, adjustment for it cannot by itself causally separate nitrogen delivery from utilization; describe this analysis as exploratory and moderate the causal wording.*

**Response.** We agree. Delivered nitrogen has been removed from the covariates of the prespecified adjusted (confirmatory) ANCOVA, which now includes only extent of resection and baseline nutritional status. The delivered-nitrogen–adjusted analysis and the utilization-efficiency analysis are now described as exploratory and descriptive, and we state explicitly that, because delivered nitrogen is a post-randomization variable largely determined by treatment allocation, adjustment for it cannot by itself causally separate nitrogen delivery from nitrogen utilization; the causal wording has been moderated and these analyses are reported as hypothesis-generating without causal claims. The dated SAP reflects this exploratory status.

We thank the editor and Reviewer 3 for these constructive comments, which have further improved the protocol.
