# Supplementary material for: The PROGAIN trial: A randomized controlled trial of high-protein peripheral parenteral nutrition on nitrogen balance and recovery after gastric cancer surgery — study protocol
Source: PLoS One. 2026 Aug 7;21(8):e0355677. doi: 10.1371/journal.pone.0355677 (PMC13450730; doi:10.1371/journal.pone.0355677)
Supplement: Peer review history

# Peer review: Decision Letter 0

**DOI:** 10.1371/journal.pone.0355677.r001

**Authors:** Athanasios Pantelis

1 Jul 2026

PONE-D-26-26899

The PROGAIN trial: a randomized controlled trial of high-protein peripheral parenteral nutrition on nitrogen balance and recovery after gastric cancer surgery — study protocol

PLOS One

Dear Dr. Song,

Thank you for submitting your manuscript to PLOS ONE. After careful consideration, we feel that it has merit but does not fully meet PLOS ONE’s publication criteria as it currently stands. Therefore, we invite you to submit a revised version of the manuscript that addresses the points raised during the review process.

Please submit your revised manuscript by Jul 31 2026 11:59PM. If you will need more time than this to complete your revisions, please reply to this message or contact the journal office at plosone@plos.org. When you're ready to submit your revision, log on to https://www.editorialmanager.com/pone/ and select the 'Submissions Needing Revision' folder to locate your manuscript file.

Please include the following items when submitting your revised manuscript:

- A letter that responds to each point raised by the academic editor and reviewer(s). You should upload this letter as a separate file labeled 'Response to Reviewers'.
- A marked-up copy of your manuscript that highlights changes made to the original version. You should upload this as a separate file labeled 'Revised Manuscript with Track Changes'.
- An unmarked version of your revised paper without tracked changes. You should upload this as a separate file labeled 'Manuscript'.

If you would like to make changes to your financial disclosure, please include your updated statement in your cover letter. Guidelines for resubmitting your figure files are available below the reviewer comments at the end of this letter.

If applicable, we recommend that you deposit your laboratory protocols in protocols.io to enhance the reproducibility of your results. Protocols.io assigns your protocol its own identifier (DOI) so that it can be cited independently in the future. For instructions see: https://journals.plos.org/plosone/s/submission-guidelines#loc-laboratory-protocols. Additionally, PLOS ONE offers an option for publishing peer-reviewed Lab Protocol articles, which describe protocols hosted on protocols.io. Read more information on sharing protocols at https://plos.org/protocols?utm_medium=editorial-email&utm_source=authorletters&utm_campaign=protocols.

As the corresponding author, your ORCID iD is verified in the submission system and will appear in the published article. PLOS supports the use of ORCID, and we encourage all coauthors to register for an ORCID iD and use it as well. Please encourage your coauthors to verify their ORCID iD within the submission system before final acceptance, as unverified ORCID iDs will not appear in the published article. *Only*  the individual author can complete the verification step; PLOS staff *cannot*  verify ORCID iDs on behalf of authors.

We look forward to receiving your revised manuscript.

Kind regards,

Athanasios G. Pantelis

Academic Editor

PLOS One

**Journal Requirements:**

When submitting your revision, we need you to address these additional requirements.

1. Please ensure that your manuscript meets PLOS ONE's style requirements, including those for file naming. The PLOS ONE style templates can be found at

https://journals.plos.org/plosone/s/file?id=wjVg/PLOSOne_formatting_sample_main_body.pdf and

https://journals.plos.org/plosone/s/file?id=ba62/PLOSOne_formatting_sample_title_authors_affiliations.pdf

2. Thank you for submitting your clinical trial to PLOS ONE and for providing the name of the registry and the registration number. The information in the registry entry suggests that your trial was registered after patient recruitment began. PLOS ONE strongly encourages authors to register all trials before recruiting the first participant in a study.

As per the journal’s editorial policy, please include in the Methods section of your paper:

1) your reasons for your delay in registering this study (after enrolment of participants started);

2) confirmation that all related trials are registered by stating: “The authors confirm that all ongoing and related trials for this drug/intervention are registered”.

3. Thank you for stating the following in the Financial Disclosure section:

“This trial is an investigator-initiated trial conceived and designed by the academic investigators. Financial support for trial conduct, restricted to the salary of a dedicated study coordinator, is provided by JW Pharmaceutical (Seoul, Republic of Korea). The funder has no role in the trial design, data collection, data analysis, data interpretation, manuscript preparation, or the decision to submit the manuscript for publication. This work was supported by the Soonchunhyang University Research Fund.”

We note that you received funding from a commercial source: JW Pharmaceutical

Please provide an amended Competing Interests Statement that explicitly states this commercial funder, along with any other relevant declarations relating to employment, consultancy, patents, products in development, marketed products, etc.

Within this Competing Interests Statement, please confirm that this does not alter your adherence to all PLOS ONE policies on sharing data and materials by including the following statement: "This does not alter our adherence to PLOS ONE policies on sharing data and materials.” (as detailed online in our guide for authors http://journals.plos.org/plosone/s/competing-interests).  If there are restrictions on sharing of data and/or materials, please state these. Please note that we cannot proceed with consideration of your article until this information has been declared.

Please include your amended Competing Interests Statement within your cover letter. We will change the online submission form on your behalf.

4. Please note that funding information should not appear in any section or other areas of your manuscript. We will only publish funding information present in the Funding Statement section of the online submission form. Please remove any funding-related text from the manuscript.

5. If the reviewer comments include a recommendation to cite specific previously published works, please review and evaluate these publications to determine whether they are relevant and should be cited. There is no requirement to cite these works unless the editor has indicated otherwise.

[Note: HTML markup is below. Please do not edit.]

Reviewers' comments:

Reviewer's Responses to Questions

**Comments to the Author**

1. Does the manuscript provide a valid rationale for the proposed study, with clearly identified and justified research questions?

The research question outlined is expected to address a valid academic problem or topic and contribute to the base of knowledge in the field.

Reviewer #1: Yes

Reviewer #2: Partly

Reviewer #3: Yes

Reviewer #4: Yes

**********

2. Is the protocol technically sound and planned in a manner that will lead to a meaningful outcome and allow testing the stated hypotheses?

The manuscript should describe the methods in sufficient detail to prevent undisclosed flexibility in the experimental procedure or analysis pipeline, including sufficient outcome-neutral conditions (e.g. necessary controls, absence of floor or ceiling effects) to test the proposed hypotheses and a statistical power analysis where applicable. As there may be aspects of the methodology and analysis which can only be refined once the work is undertaken, authors should outline potential assumptions and explicitly describe what aspects of the proposed analyses, if any, are exploratory.

Reviewer #1: Yes

Reviewer #2: Yes

Reviewer #3: No

Reviewer #4: Yes

**********

3. Is the methodology feasible and described in sufficient detail to allow the work to be replicable?

Descriptions of methods and materials in the protocol should be reported in sufficient detail for another researcher to reproduce all experiments and analyses. The protocol should describe the appropriate controls, sample size calculations, and replication needed to ensure that the data are robust and reproducible.

Reviewer #1: Yes

Reviewer #2: Yes

Reviewer #3: No

Reviewer #4: Yes

**********

4. Have the authors described where all data underlying the findings will be made available when the study is complete?

The PLOS Data policy requires authors to make all data underlying the findings described in their manuscript fully available without restriction, with rare exception, at the time of publication. The data should be provided as part of the manuscript or its supporting information, or deposited to a public repository. For example, in addition to summary statistics, the data points behind means, medians and variance measures should be available. If there are restrictions on publicly sharing data—e.g. participant privacy or use of data from a third party—those must be specified.

Reviewer #1: Yes

Reviewer #2: Yes

Reviewer #3: No

Reviewer #4: Yes

**********

5. Is the manuscript presented in an intelligible fashion and written in standard English?

PLOS ONE does not copyedit accepted manuscripts, so the language in submitted articles must be clear, correct, and unambiguous. Any typographical or grammatical errors should be corrected at revision, so please note any specific errors here.

Reviewer #1: Yes

Reviewer #2: Yes

Reviewer #3: Yes

Reviewer #4: Yes

**********

6. Review Comments to the Author

Please use the space provided to explain your answers to the questions above and, if applicable, provide comments about issues authors must address before this protocol can be accepted for publication. You may also include additional comments for the author, including concerns about research or publication ethics.

You may also provide optional suggestions and comments to authors that they might find helpful in planning their study.

(Please upload your review as an attachment if it exceeds 20,000 characters)

Reviewer #1: The design and analysis strategy are sufficiently outlined in the protocol.

PROGAIN is a single-center, prospective, parallel-group, two-arm, randomized, open-label superiority trial comparing high-protein PN with conventional PN in adults undergoing curative gastric cancer surgery, with 1:1 allocation between the investigational and control arms.

For the sample size calculation, it appears that the best available information was used. The between-group mean difference of 3 g/day in postoperative nitrogen balance was assumed, with a common standard deviation of 5 g/day. Using a two-sided significance level of 0.05 and statistical power of 80%, the minimum required sample size is 44 participants per arm (88 participants in total). Anticipating a combined withdrawal, protocol non-completion, or missing primary outcome rate of approximately 20%, the planned enrollment is increased to 55 participants per arm, giving a total target enrollment of 110 participants.

The analysis is a very simple comparison. All efficacy analyses are performed in the intention-to-treat (ITT) population, comprising all randomized participants analyzed in the group to which they were allocated. The per-protocol population is defined as participants following a more complete treatment strategy as one would expect.

The primary outcome is compared between the investigational and control arms using a two-sample t-test, with the between-group mean difference reported with a 95% 0 confidence interval. The Wilcoxon rank-sum test will be used as a sensitivity analysis if distributional assumptions are clearly violated.

All the above procedures were clearly presented in detail. Table 4 is an excellent presentation of the analysis strategy.

The data collection and quality control for this inpatient procedure is presented as required for the study. Figure 1 with the schedule of assessments and interventions is presented as well.

The overall study was well planned and presented from the statistical perspective.

Reviewer #2: This manuscript is a protocol for an investigator-initiated randomized clinical trial comparing standard versus high-protein parenteral nutrition following gastrectomy. This study will be conducted at a single center in Korea and will have a target enrollment of 110 patients. The primary outcome is nitrogen balance as of POD5, and the study was designed with 80% power to detect a mean difference of 3 g/day at two-sided alpha of 0.05.

I have a few questions, though I acknowledge that many of these will be outdated as the trial has already started accruing patients. I do look forward to the findings of the trial.

- The practice of routine parenteral nutrition, especially its use on the day before surgery, is not done in the West to my knowledge, so the findings from this trial will not really inform any practice change there. Many patients undergoing partial gastrectomy in the West have a length of stay shorter than 5 postoperative days. I wonder if the authors can comment on how often standard PN is employed in routine postoperative management, including starting on the day before surgery, in Korea and in other Asian settings.

- The authors describe relationships between nutritional status and clinical outcomes, but there is no mention of nitrogen balance as an appropriate proxy for nutritional status and protein intake, especially as it relates to outcomes. What data exist for this? Have clinically meaningful differences in nitrogen balance been established? What is the proposed significance of a nitrogen balance of 3 g/day?

- Regarding the primary endpoint selection, wouldn't it be expected that giving patients additional protein would effect a resultant change in their nitrogen balance? I'm not sure how this should inform clinical practice. It seems that selecting more relevant clinical endpoints might have been more appropriate - otherwise, who cares what the nitrogen balance is if the clinical outcomes are the same in either arm?

- Are all gastrectomy patients included (wedge, distal/subtotal, proximal, total)? Are all types of reconstruction included (Roux-en-Y, Billroth I, Billroth II, double tract)? Are all surgical approaches included (open, laparoscopic, robotic)? I would specify these somewhere in the protocol, even if this is just a blanket statement regarding all types. However, with inclusion of multiple types, the inherent nutritional risks between, for instance, total gastrectomy versus proximal gastrectomy with double-tract reconstruction would be difficult to interpret here, since the latter may be expected to derive greater benefit from their oral intake. Performing subgroup analyses on all of these patient populations may not be statistically feasible.

- How is PN handled in either group when patients experience anastomotic leak and/or develop bacteremia or fungemia?

- Will patients with underlying cirrhosis or chronic kidney disease be included? The introduction of a high-protein regimen may be risky for these patients.

- Introduction lines 41-42: Please provide citations for these outcome associations.

Reviewer #3: The PROGAIN trial protocol addresses a clinically relevant and practical question:

whether amino acid–enriched peripheral parenteral nutrition (PN) improves

postoperative nitrogen balance after curative gastrectomy, a setting in which early

oral intake is frequently insufficient and adequate protein delivery through

peripheral access alone is difficult. The protocol is clearly written and logically

structured, with appropriate attention to randomization, allocation concealment,

blinded laboratory and imaging assessment, prespecified outcomes, ethics approval,

and SPIRIT 2025 reporting. The composition table, stepwise delivery schedule, and

outcome table are detailed and helpful. There are no fatal flaws, but several

methodological and reporting issues should be resolved before publication,

principally concerning interpretation of the primary endpoint, the validity of

24-hour urinary nitrogen assessment, balance of key surgical prognostic factors, and

the retrospective registration.

MAJOR COMMENTS

1. The primary endpoint is partly determined by the intervention itself.

Nitrogen balance is calculated as nitrogen intake (oral protein plus parenteral amino

acids) minus urinary urea nitrogen and estimated non-urinary losses. Because the

intervention directly increases parenteral nitrogen intake, the primary outcome may

improve mechanically unless urinary nitrogen excretion rises proportionally. At the

POD 5 scheduled PN coverage (50%), the between-arm difference in delivered amino acids

is on the order of a few grams of nitrogen per day, so a meaningful fraction of the

assumed 3 g/day effect could be explained by intake difference alone, by definition.

The authors should state explicitly whether the question of interest is greater

protein retention per se or improved net utilization per unit of delivered nitrogen,

discuss this limitation, and avoid presenting nitrogen balance as a fully independent

biological endpoint. Please prespecify separate analyses of (a) total nitrogen intake,

(b) urinary urea nitrogen excretion, and (c) net nitrogen retention, ideally with the

delivered nitrogen included as a covariate (or an analysis of utilization efficiency).

2. The nitrogen-balance formula should use exact parenteral nitrogen delivery, not a

6.25 conversion. Table 2 provides product-specific nitrogen content per mL. Parenteral

amino acids are not equivalent to dietary protein for the 6.25 conversion. The more

precise computation is oral protein/6.25 plus the actual parenteral nitrogen delivered

from the assigned product volume. Please clarify the exact calculation used for the

primary endpoint and apply it consistently.

3. Feasibility and quality control of 24-hour urine collection require more detail.

The primary endpoint depends on a complete 24-hour urinary urea nitrogen collection on

POD 5. Under ERAS care, urinary catheters are often removed before POD 5, making

complete self-voided collection a major source of measurement noise. Please specify the

collection window, handling of missed voids, validation of completeness (e.g., urine

volume or creatinine excretion), management of discharge before POD 5, and how drain,

nasogastric, vomiting, or gastrointestinal losses are accounted for. The fixed constant

(+4 g) in the simplified Blackburn equation does not capture these GI/drain losses,

which are not negligible in this surgical population; please state this limitation.

4. The intervention is a composite, not "protein added in isolation."

The two products differ not only in amino acids but also in dextrose (70.7 vs 64.4

mg/mL), NPC/N ratio (109 vs 84), electrolytes, osmolarity, and required infusion

volume. The investigational arm therefore represents higher protein, lower

carbohydrate, and lower NPC/N simultaneously. An NPC/N of 84 is relatively low for

protein sparing and could itself modulate amino-acid utilization. The glycemic

secondary outcomes will partly reflect the difference in carbohydrate load rather than

a "high-protein" benefit. Please present the expected daily delivered amounts of amino

acids, nitrogen, glucose, lipid, electrolytes, and total volume for both arms at the

actual scheduled coverage on each POD, and discuss the composite nature of the

intervention in interpretation.

5. Randomization may not balance key surgical prognostic factors.

Stratified randomization is not used, yet a subgroup analysis by extent of resection is

planned. Extent of gastrectomy, surgical/reconstruction approach, baseline nutritional

status, diabetes, and sarcopenia can strongly influence postoperative intake,

catabolism, and nitrogen balance. Please justify the decision not to stratify, or adopt

stratified randomization if still feasible. At minimum, prespecify an adjusted primary

analysis (ANCOVA) for key prognostic covariates (baseline nutritional index, extent of

resection), which would also be more efficient than the unadjusted two-sample t-test.

6. The assumed effect size should be better justified.

The sample size assumes a 3 g/day difference with SD 5 g/day, extrapolated from studies

with different populations and routes ([12] esophagectomy observational; [13] arginine

enteral nutrition in total gastrectomy). Because scheduled PN coverage on POD 5 is only

50%, the actual difference in delivered nitrogen on the primary endpoint day may be

smaller than assumed. Please provide a quantitative rationale linking the expected POD 5

nitrogen-intake difference to the assumed 3 g/day balance difference, and comment on

sensitivity to the assumed SD and to what clinical outcome a 3 g/day difference

corresponds.

7. Missing-data handling for the primary endpoint is too vague.

Stating that multiple imputation will be "considered" above 5% missingness is

insufficient for a protocol. Please prespecify the primary estimand and the primary

missing-data strategy for incomplete POD 5 collection, early discharge, severe

complications, death, PN discontinuation, or withheld PN. Note that dropout due to

postoperative complications may not be missing-at-random; please add sensitivity

analyses under MNAR assumptions.

8. Multiplicity control for secondary outcomes is not prespecified.

Table 4 lists numerous nutritional, glycemic, complication, functional, patient-reported,

and safety outcomes. "Hierarchical testing or FDR control as appropriate" is too

nonspecific. Please distinguish key secondary from exploratory outcomes and commit to a

specific multiplicity strategy in the SAP before trial completion.

9. Blinding, outcome adjudication, and the retrospective registration.

(a) Open-label design: oral protein intake — which enters the numerator of the primary

endpoint — is recorded in 25% increments by unblinded staff/patients, leaving an

ascertainment-bias channel that the blinded UUN assay does not. Clavien–Dindo grading,

discharge readiness, ambulation, and diet tolerance are likewise susceptible. Please

clarify who adjudicates complications, whether adjudicators are blinded, and whether

standardized discharge and diet-advancement criteria are applied; state the

intake-recording limitation explicitly. (b) Registration: first randomization

13 February 2026, ClinicalTrials.gov registration 14 March 2026 (~29 days later). This

does not meet prospective-registration requirements. The authors disclose this

transparently, but please state explicitly whether any participant completed the POD 5

primary endpoint before public registration, whether the registry record fully matches

the IRB-approved protocol, and whether outcomes, eligibility, intervention schedule, or

analysis plans changed between IRB approval, first enrollment, and registration. This

is referred to the editor for a policy decision.

MINOR COMMENTS

1. Table 4 cites "Loder et al., 1989" for the nitrogen-balance formula, but this

reference is not in the reference list; the text uses Blackburn 1977 [19] and

Dickerson 2005 [20]. Please reconcile.

2. The text (p.18) states an audit of "354 inpatients," while the Table 3 footnote gives

92+160+102 = 354 measurements. Please clarify whether this is the number of patients or

of measurements.

3. In Table 3, the POD 3 early soft diet (~315 kcal/day) is lower than the POD 2 liquid

diet (~350 kcal/day). This non-monotonic intake is physiologically counterintuitive;

please verify or explain.

4. Diet-stage terminology is inconsistent ("soft diet" in text vs "congee diet" in

Table 3; "post-gastrectomy early soft diet" reads awkwardly). Please standardize.

5. POD −1 is potentially confusing; consider "preoperative day −1" or "day −1" used

consistently.

6. The introduction emphasizes that conventional PN delivers ~0.9 g/kg/day (below the

ESPEN minimum), but nitrogen balance is computed from total (oral + parenteral) protein.

Please distinguish PN-only values from total protein supply to avoid an impression of

undertreatment and concerns about equipoise.

7. Please clarify whether oral nutritional supplements are permitted during POD 1–5; if

so, how they are standardized, recorded, and incorporated into the nitrogen-balance

calculation.

8. Fig 2 legend states skeletal muscle index is measured on POD 5, whereas the Outcomes

section and Table 4 specify baseline and follow-up CT. Please correct.

9. Data availability: "no datasets generated or analyzed" conflicts with the existing

institutional audit data (S1 Table, n=354). Please adjust the wording.

10. Please specify the rationale and applicable range for the ideal-body-weight formula

(height² × 23, applied at BMI ≥25).

11. Safety: given the higher osmolarity and the manufacturer's involvement with active

recruitment, please describe peripheral line management, infusion duration, phlebitis

grading, line-replacement criteria, renal/BUN thresholds, insulin management, and

stopping rules, and justify the absence of a DSMB or describe an independent safety

review more fully.

12. The financial disclosure should include all journal-required details (grant numbers,

initials of funded authors, funder URLs where applicable).

13. In the compiled reviewer PDF, Fig 1, Fig 2, the SPIRIT checklist, and the IRB

protocol appear as file placeholders rather than embedded/readable content; please

ensure all are legible to reviewers.

OVERALL ASSESSMENT

The trial addresses an important perioperative nutrition question and the protocol is

generally well prepared. The principal issues to resolve before publication are the

interpretation of nitrogen balance as the primary endpoint (mechanical dependence on

the intervention and exact nitrogen accounting), the operational validity of 24-hour

urinary nitrogen assessment under ERAS, balance of key surgical prognostic factors, the

composite nature of the intervention, and the retrospective registration. I recommend

major revision.

Reviewer #4: This manuscript presents the study protocol of the PROGAIN randomized controlled trial, which evaluates the effect of high-protein peripheral parenteral nutrition on nitrogen balance and postoperative recovery in patients undergoing curative gastric cancer surgery. The research question addresses an important clinical gap, the trial design is rigorous, and the methodological details are thoroughly reported in full accordance with the SPIRIT 2025 statement. Core elements including ethics approval, trial registration, and statistical analysis plan are clearly and completely presented. Overall, this is a high-quality study protocol. I recommend acceptance after minor revision.

Specific Comments

1. Supplementary information on trial registration transparency

The authors have noted that the trial was registered on ClinicalTrials.gov approximately 29 days after the first participant was enrolled, attributed to administrative processing, and confirmed no substantive amendments to the core protocol. Please add a brief clarification: whether this retrospective registration has been reported to the registry platform, and further confirm that the full protocol received IRB approval before the first enrollment, and that all primary endpoints and the statistical analysis plan were finalized prior to registration without modification. This will further strengthen the credibility and transparency of the trial.

2. Rationale for the dropout rate assumption in sample size calculation

The sample size calculation assumes a 20% combined rate of withdrawal, protocol non-completion, or missing primary outcome, leading to an increase from 88 to 110 total participants. Please briefly provide the rationale for this 20% assumption — for example, by citing observed dropout rates from similar surgical nutrition trials at the same institution, or by noting that this rate aligns with typical dropout levels in comparable perioperative RCTs — to further support the sample size estimation.

3. Clarification of the unblinding procedure

The manuscript clearly describes the blinded assessment of laboratory outcomes, complication grading, and coded-group primary analysis. Please add a brief description of the unblinding process: for example, that the coded group labels (Arm A / Arm B) will be unmasked by an independent individual after database lock and finalization of the statistical analysis plan. This will make the full blinding workflow more complete and traceable.

**********

7. PLOS authors have the option to publish the peer review history of their article (what does this mean?). If published, this will include your full peer review and any attached files.

If you choose “no”, your identity will remain anonymous but your review may still be made public.

**Do you want your identity to be public for this peer review?**  For information about this choice, including consent withdrawal, please see our Privacy Policy.

Reviewer #1: No

Reviewer #2: **Yes:** Benjamin D Ferguson

Reviewer #3: No

Reviewer #4: **Yes:** He Lijian

**********

[NOTE: If reviewer comments were submitted as an attachment file, they will be attached to this email and accessible via the submission site. Please log into your account, locate the manuscript record, and check for the action link "View Attachments". If this link does not appear, there are no attachment files.]

To ensure your figures meet our technical requirements, please review our figure guidelines: https://journals.plos.org/plosone/s/figures

You may also use PLOS’s free figure tool, NAAS, to help you prepare publication quality figures: https://journals.plos.org/plosone/s/figures#loc-tools-for-figure-preparation.

NAAS will assess whether your figures meet our technical requirements by comparing each figure against our figure specifications.

# Author comment: Author response to Decision Letter 1

**DOI:** 10.1371/journal.pone.0355677.r002

7 Jul 2026

Response to Reviewers

Manuscript: PONE-D-26-26899

Title: The PROGAIN trial: a randomized controlled trial of high-protein peripheral parenteral nutrition on nitrogen balance and recovery after gastric cancer surgery — study protocol

We thank the Academic Editor and the four reviewers for their careful and constructive evaluation of our protocol. We have revised the manuscript accordingly and respond to each point below. Reviewer comments are shown in italics, followed by our response. Each reviewer’s responses are self-contained; where two reviewers raised a similar point, we provide a full answer under each. Changes are marked in the tracked-changes version of the manuscript.

Journal Requirements (Academic Editor)

1. Please ensure the manuscript meets PLOS ONE style requirements, including file naming.

Response. The manuscript, title page, and file names have been reformatted to conform to the PLOS ONE templates and file-naming conventions.

2. Registration appears to have occurred after recruitment began. Please state in the Methods (i) the reasons for the delay, and (ii) the confirmation that all related trials are registered.

Response. The Trial status section now states the reason for the delayed registration (administrative processing and access to the institutional registration system following IRB approval) and includes the required statement: “The authors confirm that all ongoing and related trials for this drug/intervention are registered.” The Trial status section additionally documents that the IRB-approved protocol and the outcomes and statistical analysis plan were finalized before the first participant was enrolled and that the registry record corresponds to that protocol.

3. You received funding from a commercial source (JW Pharmaceutical). Please provide an amended Competing Interests statement and confirm adherence to PLOS data/materials-sharing policies.

Response. The Competing Interests statement (provided in the cover letter for entry into the submission system) now explicitly names the commercial funder and reads: “This study was funded in part by JW Pharmaceutical, a commercial source, which manufactures both the investigational and comparator products. The coordinator salary and the article-processing charge were supported by JW Pharmaceutical. The funder had no role in study design, data collection and analysis, interpretation, or manuscript preparation. This does not alter our adherence to PLOS ONE policies on sharing data and materials.”

4. Funding information should not appear in the manuscript; it will be published only from the Funding Statement in the submission system.

Response. All funding-related text has been removed from the manuscript body; the funding information is provided solely in the Funding Statement of the online submission form.

5. Cite reviewer-recommended works only where relevant.

Response. We evaluated the suggested topics and added citations only where they strengthen specific statements (see Reviewer 2, Comments 1 and 7).

Reviewer #1

The design and analysis strategy are sufficiently outlined; the study is well planned and presented, and Table 4 is an excellent presentation of the analysis strategy. (No revisions requested.)

Response. We are grateful to the reviewer for the positive assessment and encouraging comments.

Reviewer #2

2-1. Routine PN, especially on the day before surgery, is not standard in the West, and Western partial-gastrectomy stays are often shorter than 5 postoperative days. Please comment on how often standard perioperative PN (including the day before surgery) is used in Korea and other Asian settings.

Response. We agree this context is important and provide the following clarification in response; because the reviewer raises this as a question and the point does not require a change to the protocol, we address it here rather than in the manuscript. Routine perioperative PN, including administration on the day before surgery, is common practice in Korea and other East Asian countries and differs from enhanced-recovery–dominant Western practice. Postoperative length of stay after gastrectomy in East Asia is substantially longer than the sub–five-day stays sometimes reported in Western partial gastrectomy: recent two-centre and multicentre analyses from Korea, China, and Japan report mean postoperative stays of approximately 8.6–9.0 days. In a Chinese multicentre ERAS cohort of 1,434 patients, the mean time to first flatus was 4.4 days and the mean time to initiation of a liquid diet was 6.1 days, indicating that adequate oral intake is typically not achieved until after POD 5. In addition, admission on the day before surgery is common in this setting for indocyanine green injection for sentinel node mapping and surgical marking, which is why PN begins on preoperative day −1. Within this context, a six-day perioperative PN course and a POD 5 nitrogen-balance endpoint fall well within the usual inpatient trajectory, while the mechanistic findings are expected to generalize beyond it. Supporting citations have been added.

2-2. There is no mention of nitrogen balance as an appropriate proxy for nutritional status/protein intake. What data support this? Have clinically meaningful differences been established? What is the significance of a 3 g/day difference?

Response. Nitrogen balance is an objective, quantifiable, blinded-assay marker of net protein metabolism — the difference between nitrogen intake and nitrogen loss — and has been used as a primary or principal endpoint in perioperative and nutritional randomized trials. It is not simply a proxy that rises automatically with intake: in the catabolic state after major surgery, additional amino acids are frequently oxidized and excreted rather than retained, and several randomized trials of enriched amino-acid regimens have shown no improvement in nitrogen balance despite greater or altered amino-acid delivery. Demonstrating an improvement therefore reflects genuine net retention rather than arithmetic. Regarding clinical meaning, a between-arm difference of 3 g/day in nitrogen balance corresponds to approximately 19 g/day of protein-equivalent and represents a clinically meaningful shift during the early postoperative catabolic period, comparable in magnitude to differences reported in prior perioperative protein-supplementation studies. To make the interpretation explicit, we have added prespecified mechanistic analyses that decompose the endpoint into total nitrogen intake, urinary urea nitrogen excretion, and net nitrogen retention, together with a nitrogen-utilization analysis. Supporting citations have been added.

2-3. Wouldn’t giving more protein necessarily change nitrogen balance? Why not select more clinically relevant endpoints?

Response. We selected nitrogen balance as the primary endpoint because it is an objective, quantifiable, blinded-assay outcome that is comparatively resistant to the ascertainment bias to which subjective or clinically graded outcomes are prone in an open-label, single-centre trial, and because it directly reflects the mechanism of the intervention (protein and nitrogen economy) on a clinically relevant short timescale; clinically oriented recovery outcomes require much larger samples to assess reliably and are captured comprehensively as prespecified secondary outcomes. We agree that a difference in nitrogen balance is meaningful only if it reflects genuine retention rather than intake alone. To address this, we have added prespecified mechanistic analyses decomposing total nitrogen intake, urinary urea nitrogen, and net retention, together with a utilization-efficiency analysis; notably, the between-arm difference in delivered nitrogen on POD 5 is only approximately 1.0–1.2 g/day, so an observed nitrogen-balance difference of the assumed 3 g/day could not be explained by intake alone and would indicate a genuine metabolic effect. The interpretation and its limitations are discussed in the revised manuscript.

2-4. Are all gastrectomy types (wedge, distal/subtotal, proximal, total), reconstructions (Roux-en-Y, Billroth I/II, double tract), and approaches (open, laparoscopic, robotic) included? Please specify, and note that heterogeneity may limit interpretation and subgroup feasibility.

Response. We have clarified eligibility. Eligibility is based on the planned extent of resection: planned total and distal (subtotal) gastrectomy are eligible, whereas planned proximal gastrectomy is excluded (now stated explicitly in Table 1). All standard reconstructions (Billroth I, Billroth II, Roux-en-Y) and surgical approaches (open, laparoscopic, robotic) are permitted. If the actual procedure differs from the plan (including an unplanned proximal gastrectomy), the participant is retained and analyzed in the allocated arm under the intention-to-treat principle, with the actual procedure recorded and examined in per-protocol/sensitivity analyses. We have added a statement acknowledging that heterogeneity of resection type may complicate interpretation and that subgroup analyses are exploratory and may be underpowered.

2-5. How is PN handled when patients develop anastomotic leak and/or bacteremia or fungemia?

Response. For severe postoperative complications, including anastomotic leak, bacteremia, or fungemia, the allocated PN is discontinued at the discretion of the treating clinician; such participants are retained in the intention-to-treat population, excluded from the per-protocol population, and flagged for sensitivity analysis. This is now stated in the Interventions (Monitoring and dose modification) and Safety sections.

2-6. Will patients with cirrhosis or chronic kidney disease be included, given the risks of a high-protein regimen?

Response. Patients with significant hepatic or renal impairment are excluded by the existing screening thresholds (AST or ALT > 3× ULN, total bilirubin > 3× ULN, serum creatinine > 2× ULN), which exclude decompensated hepatic disease and clinically significant chronic kidney disease. Hepatic and renal function, blood urea nitrogen, glucose, and electrolytes are monitored daily during the intervention, with predefined safety triggers for dose reduction or discontinuation. We have made this explicit in the Eligibility and Safety sections.

2-7. Introduction (lines 41–42): please provide citations for the stated outcome associations.

Response. Citations have been added for the associations between inadequate perioperative protein supply and delayed wound healing, infectious complications, prolonged hospital stay (perioperative nutrition literature), and impaired adjuvant chemotherapy tolerance (our prior institutional data).

Reviewer #3 — Major comments

1. The primary endpoint is partly determined by the intervention itself; a fraction of the assumed effect could be explained by intake difference alone. State the question of interest (retention vs utilization), discuss this limitation, and prespecify separate analyses of (a) total nitrogen intake, (b) urinary urea nitrogen, and (c) net nitrogen retention, ideally with delivered nitrogen as a covariate or a utilization-efficiency analysis.

Response. We thank the reviewer for this important point, which we have addressed in three ways. First, we have clarified the interpretation: nitrogen balance is presented as a clinically oriented marker of net nitrogen retention that is, by design, partly dependent on nitrogen intake, rather than as a fully independent biological endpoint. Second, we emphasize that an improvement is not a foregone conclusion: in catabolic surgical patients, additional amino acids are frequently oxidized and excreted rather than retained, and several randomized trials of enriched amino-acid regimens have found no improvement in nitrogen balance; the trial therefore tests whether this formulation produces genuine net retention. Third, we have added prespecified mechanistic secondary analyses that decompose the endpoint into (a) total nitrogen intake, (b) urinary urea nitrogen excretion, and (c) net nitrogen retention, together with a nitrogen-utilization analysis expressed as net retention relative to delivered nitrogen and, equivalently, as an analysis of the primary endpoint adjusting for delivered nitrogen as a covariate (integrated into the ANCOVA described in Major 5). Because the between-arm difference in delivered nitrogen on POD 5 is only about 1.0–1.2 g/day (see Major 6), an observed nitrogen-balance difference of the assumed magnitude could not be explained by intake alone and would indicate a genuine difference in retention/utilization. A corresponding limitation has been added.

2. The nitrogen-balance formula should use exact parenteral nitrogen delivery, not a 6.25 conversion; Table 2 provides product-specific nitrogen content.

Response. We agree and have revised the formula. Nitrogen balance is now computed as [oral protein (g) ÷ 6.25 + parenteral nitrogen delivered (g)] − (urinary urea nitrogen (g) + 4), where parenteral nitrogen is derived directly from the product-specific nitrogen content in Table 2 (5.21 mg/mL for the control and 6.17 mg/mL for the investigational product) multiplied by the administered volume, rather than by dividing amino-acid grams by 6.25. The 6.25 factor is retained only for dietary (oral) protein. This exact accounting is applied consistently and underlies the mechanistic decomposition in Major 1.

3. Feasibility and quality control of the 24-hour urine collection require detail: collection window, missed voids, completeness validation, discharge before POD 5, and drain/NG/GI losses; note the +4 g constant limitation.

Response. We have added operational detail. The 24-hour urinary urea nitrogen collection is performed over POD 5 (00:00–24:00) by self-voided collection, as urinary catheters are removed early under the enhanced-recovery pathway; participants receive standardized instruction (discarding the first morning void and including the final void at hour 24), and collection start and end times are documented. Total 24-hour urine volume, which is recorded for every participant, is used as a plausibility check, and any missed or incomplete void is flagged; collections judged incomplete are handled per the missing-data plan (Major 7). Because all participants remain hospitalized through POD 5 in this population, discharge before the primary assessment is not anticipated. Participants with clinically significant non-urinary gastrointestinal losses (high-output drainage, prolonged nasogastric aspiration, substantial vomiting, or anastomotic leak) are flagged and examined in a prespecified sensitivity analysis. We have added a limitation stating that the fixed +4 g constant does not capture surgical gastrointestinal losses and that completeness was not verified by creatinine excretion; because both apply similarly across the randomized arms, they are expected to affect the absolute nitrogen balance more than the between-arm comparison, and residual collection error is expected to be non-differential.

4. The intervention is a composite (amino acids, dextrose, NPC/N, electrolytes, osmolarity, and volume all differ). Present the expected daily delivered amounts for both arms at the scheduled coverage on each POD, and discuss the composite nature.

Response. We agree. A new supporting table (S_ Table) presents the expected daily delivered amounts of amino acids, nitrogen, glucose, and lipid (with total volume) for both arms at the scheduled coverage on each intervention day (POD −1 to POD 5); electrolytes scale proportionally with volume, as noted in the table footnote. The Discussion now states explicitly that the investigational formulation simultaneously delivers higher protein, lower carbohydrate, and a lower NPC/N ratio, and that the glycemic secondary outcomes therefore partly reflect the difference in carbohydrate load rather than a high-protein effect alone; this compositional rationale was intentional and is now made explicit.

5. Stratified randomization is not used, yet a subgroup analysis by extent of resection is planned. Justify non-stratification or adopt it; at minimum prespecify an adjusted (ANCOVA) primary analysis for key prognostic covariates.

Response. We appreciate this comment and have both clarified and strengthened the analysis

Attachment Submitted filename: Response_to_Reviewers_PROGAIN.docx

# Peer review: Decision Letter 1

**DOI:** 10.1371/journal.pone.0355677.r003

**Authors:** Athanasios Pantelis

20 Jul 2026

PONE-D-26-26899R1

The PROGAIN trial: a randomized controlled trial of high-protein peripheral parenteral nutrition on nitrogen balance and recovery after gastric cancer surgery — study protocol

PLOS One

Dear Dr. Song,

Thank you for submitting your manuscript to PLOS ONE. After careful consideration, we feel that it has merit but does not fully meet PLOS ONE’s publication criteria as it currently stands. Therefore, we invite you to submit a revised version of the manuscript that addresses the points raised during the review process.

==============================

**ACADEMIC EDITOR:**  Please address the reviewer's minor revision comments before proceeding with the final submission.

==============================

Please submit your revised manuscript by Aug 04 2026 11:59PM. If you will need more time than this to complete your revisions, please reply to this message or contact the journal office at plosone@plos.org. When you're ready to submit your revision, log on to https://www.editorialmanager.com/pone/ and select the 'Submissions Needing Revision' folder to locate your manuscript file.

Please include the following items when submitting your revised manuscript:

- A letter that responds to each point raised by the academic editor and reviewer(s). You should upload this letter as a separate file labeled 'Response to Reviewers'.
- A marked-up copy of your manuscript that highlights changes made to the original version. You should upload this as a separate file labeled 'Revised Manuscript with Track Changes'.
- An unmarked version of your revised paper without tracked changes. You should upload this as a separate file labeled 'Manuscript'.

If you would like to make changes to your financial disclosure, please include your updated statement in your cover letter. Guidelines for resubmitting your figure files are available below the reviewer comments at the end of this letter.

If applicable, we recommend that you deposit your laboratory protocols in protocols.io to enhance the reproducibility of your results. Protocols.io assigns your protocol its own identifier (DOI) so that it can be cited independently in the future. For instructions see: https://journals.plos.org/plosone/s/submission-guidelines#loc-laboratory-protocols. Additionally, PLOS ONE offers an option for publishing peer-reviewed Lab Protocol articles, which describe protocols hosted on protocols.io. Read more information on sharing protocols at https://plos.org/protocols?utm_medium=editorial-email&utm_source=authorletters&utm_campaign=protocols.

As the corresponding author, your ORCID iD is verified in the submission system and will appear in the published article. PLOS supports the use of ORCID, and we encourage all coauthors to register for an ORCID iD and use it as well. Please encourage your coauthors to verify their ORCID iD within the submission system before final acceptance, as unverified ORCID iDs will not appear in the published article. *Only*  the individual author can complete the verification step; PLOS staff *cannot*  verify ORCID iDs on behalf of authors.

We look forward to receiving your revised manuscript.

Kind regards,

Athanasios G. Pantelis

Academic Editor

PLOS One

Journal Requirements:

If the reviewer comments include a recommendation to cite specific previously published works, please review and evaluate these publications to determine whether they are relevant and should be cited. There is no requirement to cite these works unless the editor has indicated otherwise.

Please review your reference list to ensure that it is complete and correct. If you have cited papers that have been retracted, please include the rationale for doing so in the manuscript text, or remove these references and replace them with relevant current references. Any changes to the reference list should be mentioned in the rebuttal letter that accompanies your revised manuscript. If you need to cite a retracted article, indicate the article’s retracted status in the References list and also include a citation and full reference for the retraction notice.

[Note: HTML markup is below. Please do not edit.]

Reviewers' comments:

Reviewer's Responses to Questions

**Comments to the Author**

1. Does the manuscript provide a valid rationale for the proposed study, with clearly identified and justified research questions?

The research question outlined is expected to address a valid academic problem or topic and contribute to the base of knowledge in the field.

Reviewer #1: Yes

Reviewer #2: Yes

Reviewer #3: Yes

Reviewer #4: Yes

**********

2. Is the protocol technically sound and planned in a manner that will lead to a meaningful outcome and allow testing the stated hypotheses?

The manuscript should describe the methods in sufficient detail to prevent undisclosed flexibility in the experimental procedure or analysis pipeline, including sufficient outcome-neutral conditions (e.g. necessary controls, absence of floor or ceiling effects) to test the proposed hypotheses and a statistical power analysis where applicable. As there may be aspects of the methodology and analysis which can only be refined once the work is undertaken, authors should outline potential assumptions and explicitly describe what aspects of the proposed analyses, if any, are exploratory.

Reviewer #1: Yes

Reviewer #2: Yes

Reviewer #3: Yes

Reviewer #4: Yes

**********

3. Is the methodology feasible and described in sufficient detail to allow the work to be replicable?

Descriptions of methods and materials in the protocol should be reported in sufficient detail for another researcher to reproduce all experiments and analyses. The protocol should describe the appropriate controls, sample size calculations, and replication needed to ensure that the data are robust and reproducible.

Reviewer #1: Yes

Reviewer #2: Yes

Reviewer #3: Yes

Reviewer #4: Yes

**********

4. Have the authors described where all data underlying the findings will be made available when the study is complete?

The PLOS Data policy requires authors to make all data underlying the findings described in their manuscript fully available without restriction, with rare exception, at the time of publication. The data should be provided as part of the manuscript or its supporting information, or deposited to a public repository. For example, in addition to summary statistics, the data points behind means, medians and variance measures should be available. If there are restrictions on publicly sharing data—e.g. participant privacy or use of data from a third party—those must be specified.

Reviewer #1: Yes

Reviewer #2: Yes

Reviewer #3: Yes

Reviewer #4: Yes

**********

5. Is the manuscript presented in an intelligible fashion and written in standard English?

PLOS ONE does not copyedit accepted manuscripts, so the language in submitted articles must be clear, correct, and unambiguous. Any typographical or grammatical errors should be corrected at revision, so please note any specific errors here.

Reviewer #1: Yes

Reviewer #2: Yes

Reviewer #3: Yes

Reviewer #4: Yes

**********

6. Review Comments to the Author

Please use the space provided to explain your answers to the questions above and, if applicable, provide comments about issues authors must address before this protocol can be accepted for publication. You may also include additional comments for the author, including concerns about research or publication ethics.

You may also provide optional suggestions and comments to authors that they might find helpful in planning their study.

(Please upload your review as an attachment if it exceeds 20,000 characters)

Reviewer #1: My comments have been addressed.

XXXXXXXXXXXXXXXXXXXXXXXXXXXXXXXXXXXXXXXXXXXXXXXXXXXXXXXXXXXXXXXXXXXXXXXXXXXXXXXXXXXXXXXXXXXXXXXXXXXXXXXXXXXXXXXXXXXXXXXXXXXXXXXXXXXXXXXXXXXXXXXXXXXXXXXXXXXXXXXXX

Reviewer #2: Thank you to the authors for their responses and updates to the manuscript, which has been strengthened in the process.

Reviewer #3: The authors have carefully addressed the reviewers’ comments, and the revised protocol has been substantially improved. Most of the substantive methodological concerns have been resolved. However, several points should be corrected or clarified before acceptance.

1. The manuscript states that protocol version 1.2, which changes the randomization description from stratified to non-stratified block randomization, remains under IRB review. Please provide the final IRB approval and upload the approved version 1.2 together with its English translation. Please also clarify how many participants were randomized before approval of this amendment and how this discrepancy was formally handled.

2. The footnote to Table 4 remains inconsistent with the revised Statistical Analysis section. It still refers to hierarchical testing or false-discovery-rate control and to multiple imputation only when missingness exceeds 5%. Please revise it to reflect the Holm procedure for the two key secondary outcomes, the primary MAR-based multiple-imputation strategy, and the MNAR tipping-point sensitivity analysis. The two key secondary outcomes and the exact definition and timepoint of “early change in prealbumin” should also be identified in Table 4.

3. Please clarify the dates and version numbers of the amended protocol and statistical analysis plan incorporating the ANCOVA, mechanistic nitrogen analyses, multiplicity procedure, and missing-data analyses. These analyses should not be described simply as “prespecified” unless they are documented in a dated protocol or SAP. Please confirm that they were finalized before any aggregate or between-group analysis.

4. The Data Sharing Statement says that no participant outcome datasets have been generated, whereas the Trial Status section states that confirmed POD 5 results were already available for three participants. Please revise this to clarify that outcome data are being collected but that no aggregate or treatment-group analysis has been performed.

5. Please correct the remaining internal and editorial inconsistencies, including “congee diet” in Table 3, the extraneous citation “[9]” at line 196, and inconsistent citation formatting. The rationale for the 20% sample-size inflation should relate specifically to missingness of the POD 5 primary outcome rather than attrition during the 12-month follow-up.

6. Because delivered nitrogen is a post-randomization variable largely determined by treatment allocation, adjustment for it cannot by itself causally separate the effects of nitrogen delivery from nitrogen utilization. This analysis should be described as exploratory, and the causal wording should be moderated.

Subject to these revisions, I believe the protocol will be suitable for publication.

Reviewer #4: The authors have provided detailed, point-by-point responses to all comments raised by the Academic Editor and the four reviewers, and have completed high-quality revisions to the manuscript. Key methodological concerns — including refinement of the nitrogen balance formula, specification of the estimand and missing data strategy, multiplicity control, clarification of trial registration transparency, discussion of the composite intervention nature, and mitigation of open-label bias — have all been addressed with substantial additions and clearly prespecified analysis plans. The rigor, standardization, and transparency of the study protocol have been notably improved.

The revised protocol is scientifically sound and fully reported, in compliance with the SPIRIT 2025 statement and the publication standards of PLOS ONE. No remaining academic issues require further revision. I recommend acceptance of this manuscript for publication.

**********

7. PLOS authors have the option to publish the peer review history of their article (what does this mean?). If published, this will include your full peer review and any attached files.

If you choose “no”, your identity will remain anonymous but your review may still be made public.

**Do you want your identity to be public for this peer review?**  For information about this choice, including consent withdrawal, please see our Privacy Policy.

Reviewer #1: No

Reviewer #2: **Yes:** Ben Ferguson

Reviewer #3: No

Reviewer #4: No

**********

[NOTE: If reviewer comments were submitted as an attachment file, they will be attached to this email and accessible via the submission site. Please log into your account, locate the manuscript record, and check for the action link "View Attachments". If this link does not appear, there are no attachment files.]

To ensure your figures meet our technical requirements, please review our figure guidelines: https://journals.plos.org/plosone/s/figures

You may also use PLOS’s free figure tool, NAAS, to help you prepare publication quality figures: https://journals.plos.org/plosone/s/figures#loc-tools-for-figure-preparation.

NAAS will assess whether your figures meet our technical requirements by comparing each figure against our figure specifications.

# Author comment: Author response to Decision Letter 2

**DOI:** 10.1371/journal.pone.0355677.r004

21 Jul 2026

Response to Reviewers (Revision 2)

Manuscript: PONE-D-26-26899R1

Title: The PROGAIN trial: a randomized controlled trial of high-protein peripheral parenteral nutrition on nitrogen balance and recovery after gastric cancer surgery — study protocol

We thank the Academic Editor and the reviewers for the positive assessment. Reviewers 1, 2, and 4 indicated that their comments were fully addressed, and Reviewer 4 recommended acceptance. Reviewer 3 raised six remaining points for clarification, which we address below. Reviewer comments are in italics; changes are marked in the tracked-changes manuscript.

Academic Editor

Please address the reviewer’s minor revision comments before final submission.

Response. We have addressed all six of Reviewer 3’s points, as detailed below. No changes to the financial disclosure are made; the cover letter and submission-system statements are unchanged.

Reviewer #1, #2, and #4

Response. We are grateful for the confirmation that our previous revisions resolved the reviewers’ comments and for Reviewer 4’s recommendation of acceptance. No further changes were requested by these reviewers.

Reviewer #3

1. Protocol v1.2 (stratified → non-stratified randomization) remains under IRB review. Provide the final IRB approval and the approved v1.2 with English translation, and clarify how many participants were randomized before this amendment and how the discrepancy was formally handled.

Response. Protocol amendment version 1.2 was approved by the Institutional Review Board of Soonchunhyang University Cheonan Hospital on 16 July 2026 (SCHCA IRB 2025-11-024-003). We now provide the IRB approval notice (Korean original with an English translation) as an “Other” file, and the approved protocol version 1.2 with its English translation as S1 Protocol. We have also clarified the conduct: the trial has used non-stratified block randomization consistently from the first participant, and all participants randomized to date were allocated by this single, unchanged method. The protocol document had described stratified randomization; the amendment therefore corrects the protocol document to match the actual, unchanged conduct rather than changing the randomization method, and it affects neither eligibility, the intervention, the endpoints, nor the target sample size. The Trial status section has been updated accordingly.

2. The Table 4 footnote is inconsistent with the revised Statistical Analysis section (still refers to hierarchical/FDR and to MI only when missingness exceeds 5%). Revise it to reflect the Holm procedure, the MAR-based MI strategy, and the MNAR tipping-point analysis, and identify the two key secondary outcomes and the exact definition and timepoint of “early change in prealbumin” in Table 4.

Response. The Table 4 footnote has been rewritten to match the Statistical Analysis section. It now states that the single primary endpoint is the sole confirmatory analysis; that the two key secondary endpoints — POD 3 nitrogen balance and the early change in serum prealbumin, defined as the change from the preoperative (POD −1) baseline to POD 5 — are controlled for family-wise error by the Holm procedure independently of the primary result; and that the primary-endpoint missing data are handled by MAR-based multiple imputation with complete-case and MNAR delta-adjusted (tipping-point) sensitivity analyses. The two key secondary outcomes and the prealbumin definition/timepoint are now identified in the footnote.

3. Clarify the dates and version numbers of the amended protocol and SAP incorporating the ANCOVA, mechanistic analyses, multiplicity procedure, and missing-data analyses; these should not be described simply as “prespecified” unless documented in a dated protocol or SAP, finalized before any aggregate or between-group analysis.

Response. We have prepared a dated Statistical Analysis Plan (SAP version 1.0, 21 July 2026) documenting the covariate-adjusted ANCOVA, the exploratory mechanistic nitrogen analyses, the multiplicity procedure, and the missing-data strategy; it is provided as a supporting file (S2 File) and is cited in the Trial status section and the Table 4 footnote. The SAP states, and we confirm, that it was finalized before any aggregate or between-group analysis of trial data (the trial is recruiting and no such analysis has been performed). The covariate-adjusted ANCOVA is additionally documented in the IRB-approved protocol version 1.2 (approved 16 July 2026). The manuscript now cites the dated SAP and protocol version so that these analyses are supported as genuinely prespecified.

4. The Data Sharing Statement says no participant outcome datasets have been generated, whereas the Trial Status section states that confirmed POD 5 results were available for three participants. Revise to clarify that outcome data are being collected but no aggregate or treatment-group analysis has been performed.

Response. The Data Sharing Statement has been revised to read that participant outcome data are being collected in this ongoing trial but that no aggregate or treatment-group analysis has been performed, resolving the inconsistency with the Trial status section.

5. Correct remaining inconsistencies: “congee diet” in Table 3, the extraneous citation “[9]” at line 196, inconsistent citation formatting, and the 20% sample-size inflation rationale (should relate to missingness of the POD 5 primary outcome, not 12-month attrition).

Response. All items have been corrected. “Congee diet” in Table 3 is now “soft diet,” consistent with the standardized terminology. The extraneous “[9]” has been removed. Citation formatting has been unified to the numeric style throughout, including the nitrogen-balance formula citation in Table 4 (now numeric). The rationale for the 20% inflation has been rewritten to relate specifically to missingness of the POD 5 primary outcome (incomplete 24-hour collection, early events, PN discontinuation, or death before POD 5) rather than to attrition during the 12-month follow-up.

6. Because delivered nitrogen is a post-randomization variable largely determined by allocation, adjustment for it cannot by itself causally separate nitrogen delivery from utilization; describe this analysis as exploratory and moderate the causal wording.

Response. We agree. Delivered nitrogen has been removed from the covariates of the prespecified adjusted (confirmatory) ANCOVA, which now includes only extent of resection and baseline nutritional status. The delivered-nitrogen–adjusted analysis and the utilization-efficiency analysis are now described as exploratory and descriptive, and we state explicitly that, because delivered nitrogen is a post-randomization variable largely determined by treatment allocation, adjustment for it cannot by itself causally separate nitrogen delivery from nitrogen utilization; the causal wording has been moderated and these analyses are reported as hypothesis-generating without causal claims. The dated SAP reflects this exploratory status.

We thank the editor and Reviewer 3 for these constructive comments, which have further improved the protocol.

Attachment Submitted filename: Response_to_Reviewers_PROGAIN_R2.docx

# Editor report: Decision Letter 2

**DOI:** 10.1371/journal.pone.0355677.r005

**Authors:** Athanasios Pantelis

26 Jul 2026

The PROGAIN trial: a randomized controlled trial of high-protein peripheral parenteral nutrition on nitrogen balance and recovery after gastric cancer surgery — study protocol

PONE-D-26-26899R2

Dear Dr. Song,

We’re pleased to inform you that your manuscript has been judged scientifically suitable for publication and will be formally accepted for publication once it meets all outstanding technical requirements.

Within one week, you’ll receive an e-mail detailing the required amendments. When these have been addressed, you’ll receive a formal acceptance letter and your manuscript will be scheduled for publication.

An invoice will be generated when your article is formally accepted. Please note, if your institution has a publishing partnership with PLOS and your article meets the relevant criteria, all or part of your publication costs will be covered. Please make sure your user information is up-to-date by logging into Editorial Manager at Editorial Manager® and clicking the ‘Update My Information' link at the top of the page. For questions related to billing, please contact billing support.

If your institution or institutions have a press office, please notify them about your upcoming paper to help maximize its impact. If they’ll be preparing press materials, please inform our press team as soon as possible -- no later than 48 hours after receiving the formal acceptance. Your manuscript will remain under strict press embargo until 2 pm Eastern Time on the date of publication. For more information, please contact onepress@plos.org.

Kind regards,

Athanasios G. Pantelis

Academic Editor

PLOS One

Additional Editor Comments (optional):

All reviewers' comments have been effectively and adequately addressed. Congratulations to the authors for their effort.

Reviewers' comments:

# Editor report: Acceptance letter

**DOI:** 10.1371/journal.pone.0355677.r006

**Authors:** Athanasios Pantelis

PONE-D-26-26899R2

PLOS One

Dear Dr. Song,

I'm pleased to inform you that your manuscript has been deemed suitable for publication in PLOS One. Congratulations! Your manuscript is now being handed over to our production team.

At this stage, our production department will prepare your paper for publication. This includes ensuring the following:

* All references, tables, and figures are properly cited

* All relevant supporting information is included in the manuscript submission,

* There are no issues that prevent the paper from being properly typeset

You will receive further instructions from the production team, including instructions on how to review your proof when it is ready. Please keep in mind that we are working through a large volume of accepted articles, so please give us a few days to review your paper and let you know the next and final steps.

Lastly, if your institution or institutions have a press office, please let them know about your upcoming paper now to help maximize its impact. If they'll be preparing press materials, please inform our press team within the next 48 hours. Your manuscript will remain under strict press embargo until 2 pm Eastern Time on the date of publication. For more information, please contact onepress@plos.org.

You will receive an invoice from PLOS for your publication fee after your manuscript has reached the completed accept phase. If you receive an email requesting payment before acceptance or for any other service, this may be a phishing scheme. Learn how to identify phishing emails and protect your accounts at https://explore.plos.org/phishing.

If we can help with anything else, please email us at customercare@plos.org.

Thank you for submitting your work to PLOS ONE and supporting open access.

Kind regards,

PLOS ONE Editorial Office Staff

on behalf of

Dr. Athanasios G. Pantelis

Academic Editor

PLOS One
